# The Preventive Effect of Exercise and Oral Branched-Chain Amino Acid Supplementation on Obesity-Induced Brain Changes in Ldlr−/−.Leiden Mice

**DOI:** 10.3390/nu15071716

**Published:** 2023-03-31

**Authors:** Klara J. Lohkamp, Anita M. van den Hoek, Gemma Solé-Guardia, Maria Lisovets, Talissa Alves Hoffmann, Konstantina Velanaki, Bram Geenen, Vivienne Verweij, Martine C. Morrison, Robert Kleemann, Maximilian Wiesmann, Amanda J. Kiliaan

**Affiliations:** 1 Department of Medical Imaging, Anatomy, Radboud University Medical Center, Donders Institute for Brain, Cognition, and Behavior, Preclinical Imaging Center PRIME, Radboud Alzheimer Center, 6525 EZ Nijmegen, The Netherlands; klara.lohkamp@radboudumc.nl (K.J.L.); gemma.soleguardia@radboudumc.nl (G.S.-G.); m.lisovets@amsterdamumc.nl (M.L.); talissaalveshoffmann@outlook.com (T.A.H.); konstantina.velanaki@ru.nl (K.V.); bram.geenen@radboudumc.nl (B.G.); vivienne.verweij@radboudumc.nl (V.V.); maximilian.wiesmann@radboudumc.nl (M.W.); 2 Department of Metabolic Health Research, Netherlands Organisation for Applied Scientific Research (TNO), 2333 BE Leiden, The Netherlands; a.vandenhoek@tno.nl (A.M.v.d.H.); martine.morrison@tno.nl (M.C.M.); robert.kleemann@tno.nl (R.K.)

**Keywords:** obesity, branched-chain amino acids, MRI, mouse model, dietary treatment, exercise

## Abstract

Exercise and dietary interventions are promising approaches to tackle obesity and its obesogenic effects on the brain. We investigated the impact of exercise and possible synergistic effects of exercise and branched-chain amino acids (BCAA) supplementation on the brain and behavior in high-fat-diet (HFD)-induced obese Ldlr−/−.Leiden mice. Baseline measurements were performed in chow-fed Ldlr−/−.Leiden mice to assess metabolic risk factors, cognition, and brain structure using magnetic resonance imaging. Thereafter, a subgroup was sacrificed, serving as a healthy reference. The remaining mice were fed an HFD and divided into three groups: (i) no exercise, (ii) exercise, or (iii) exercise and dietary BCAA. Mice were followed for 6 months and aforementioned tests were repeated. We found that exercise alone changed cerebral blood flow, attenuated white matter loss, and reduced neuroinflammation compared to non-exercising HFD-fed mice. Contrarily, no favorable effects of exercise on the brain were found in combination with BCAA, and neuroinflammation was increased. However, cognition was slightly improved in exercising mice on BCAA. Moreover, BCAA and exercise increased the percentage of epididymal white adipose tissue and muscle weight, decreased body weight and fasting insulin levels, improved the circadian rhythm, and transiently improved grip strength. In conclusion, BCAA should be supplemented with caution, although beneficial effects on metabolism, behavior, and cognition were observed.

## 1. Introduction

Obesity (body mass index of ≥30) is a global health problem with a broad set of comorbidities that increase the risk of metabolic and cardiovascular diseases [1]. Subsequently, obesity has been linked to structural and functional brain changes, cognitive impairment, and dementia [2,3]. Neuroimaging studies have revealed that obesity is associated with lower cerebral blood flow (CBF), reduced white matter (WM) and grey matter (GM) integrity, and changes in functional connectivity [4,5,6,7]. One of the primary drivers of obesity is the excessive consumption of diets high in saturated fats, coupled with a sedentary lifestyle [8]. Hence, more research focused on preventive treatments, targeting physical activity and dietary modifications, is urgently needed.

While the impact of obesity on the cardiovascular system and metabolism has been extensively studied, the exact mechanisms underlying the detrimental effects of obesity on the brain are not yet fully understood. Obesity leads to a decline in cerebrovascular health due to various risk factors such as dyslipidemia, hyperinsulinemia, and hyperglycemia, often resulting in hypertension and atherosclerosis [9,10,11]. Vascular dysfunction, in turn, is known to exacerbate vascular inflammation and microglial activation in the brain [12]. In addition, white adipose tissue (WAT) in obesity secretes pro-inflammatory cytokines, which cause systemic inflammation and damage to the microvasculature of the brain, as observed in early pathology of aging and dementia [13,14]. Markers for endothelial cells and vascular health, such as glucose transporter 1 (GLUT-1), have been reported to be decreased in HFD-fed mice [15]. Therefore, available evidence points towards inflammation and other vascular dysfunctions as possible pathways responsible for structural and functional brain changes in obesity [16].

Clinical and pre-clinical studies have shown that physical exercise is a ‘polypill’ to prevent or treat the majority of the aforementioned comorbidities of obesity (e.g., hypertension, insulin resistance, dyslipidemia, and inflammation) [17,18,19,20,21]. In healthy humans, several studies have shown the benefits of physical exercise on brain health by improving CBF, preserving WM integrity, increasing the size of the hippocampus, and subsequently improving cognition [22,23,24,25]. Only a few studies have investigated the effect of exercise on neuroimaging parameters in chronic obesity. In overweight children, exercise improved WM integrity in the frontal and temporal lobes [26], and promoted greater refinement of resting-state networks [27]. More recently, studies using animal models of obesity have attempted to investigate the influence of exercise interventions on potential underlying physiological pathways. Herein, exercise was found to protect against obesity-associated cognitive dysfunction by improving hippocampal insulin signaling and neurogenesis [28]. Moreover, exercise prevented cerebrovascular and WM damage by improving myelin turnover in western-diet-fed mice [29]. However, detailed neuroimaging studies, focusing on the efficacy of preventing or treating obesity-related brain changes, are lacking.

Over the past decade, oral supplementation with branched-chain amino acids (BCAA; valine, leucine, and isoleucine) has gained popularity among athletes. BCAA are essential amino acids that are uniquely extrahepatically oxidized in the skeletal muscle, where their metabolites contribute to energy metabolism and protein synthesis [30], potentially reducing muscle damage and promoting muscle recovery [31]. In addition to their effects on skeletal muscle, BCAA also influence physiological and metabolic processes such as adipogenesis [32], immune function [33], and pancreatic insulin secretion [34]. In an HFD-induced mouse model of obesity, BCAA improved glucose tolerance, reduced hepatic steatosis, and attenuated adipose tissue inflammation [35]. Several studies suggest that BCAA may affect the hormonal regulators of satiety, resulting in decreased appetite after BCAA consumption [36]. In addition, BCAA can directly cross the blood–brain barrier via the same carrier systems as tryptophan—the precursor of serotonin [37]. Increased plasma BCAA concentrations reduce tryptophan uptake, and thereby serotonin synthesis, due to competition for the same transporter, which may reduce central fatigue during exercise [37]. Nevertheless, the exact mechanisms of BCAA metabolism in the brain as well as the synergistic capacity of BCAA in combination with exercise remain elusive.

We propose that a combination of exercise and a BCAA-rich diet may be beneficial in preventing obesity-induced brain changes, either by enhancing the effects of exercise or via metabolic pathways [38].

The present animal study has multiple objectives. We set out to investigate the effect of voluntary exercise as well as the possible synergistic effect of voluntary exercise in combination with oral BCAA supplementation on HFD-induced brain changes, subsequent behavioral alterations, and underlying physiological pathways in a longitudinal study (6 months). The choice of a longitudinal study design allowed us to study the temporal sequence of pathological brain changes induced by chronic obesity, while at the same time determining the efficacy of daily lifestyle modifications in mitigating the negative effects of obesity across a wide age range and at different stages of disease severity. We used low-density lipoprotein receptor knock-out Leiden mice (Ldlr−/−.Leiden), an HFD-induced mouse model of obesity that develops obesity-associated dysfunctions, such as WAT inflammation, insulin resistance, atherosclerosis, and hypertension [39,40,41,42,43,44,45,46,47,48]. As a result, this model exhibits abnormal small vessel morphology, endothelial dysfunction, blood–brain-barrier leakage, impaired cerebral blood flow, white matter loss, decreased functional connectivity, neuroinflammation, and cognitive impairment, which closely resemble the progression of human small vessel disease leading to dementia [40,41,49,50,51,52,53]. Using state-of-the-art magnetic resonance imaging (MRI) techniques, we assessed treatment effects on CBF and microstructural changes in GM and WM, as well as temporal changes in functional connectivity in the brain. The neuroimaging experiments were paralleled by an extensive behavioral and cognitive test battery to closely monitor treatment effects on cognition, motor function, and animal activity. In addition, we quantified myelin density and quality using polarized light imaging (PLI) and assessed vascular integrity and neuroinflammation using immunohistochemistry. 

## 2. Materials and Methods

### 2.1. Animals, Diets, and Study Design

This animal study was approved by the Central Authority for Scientific Procedures on Animals (CCD, The Hague, the Netherlands, approval number: AVD5010020172064) and ethically approved by the TNO Animal Welfare Body (TNO, the Netherlands; Permit number: TNO-476) and the Veterinary Authority of the Radboud university medical center (Nijmegen, the Netherlands; approval number: 2020-0033-001). The study was performed and reported according to the ARRIVE guidelines [54].

In total, 64 male Ldlr−/−.Leiden mice, from a specified pathogen-free breeding stock at the AAALAC-accredited animal facility at TNO Metabolic Health Research (Leiden, the Netherlands), were used in this study. At 2 months of age, mice with the lowest glucose levels after 5 h of fasting were excluded from the study (*n* = 6). Low glucose values are indicative of abnormal liver function leading to a higher risk of developing fibrosis. The remaining 58 animals were shipped to the Preclinical Imaging Center (PRIME) at the Animal Research Facility, Radboudumc Nijmegen, the Netherlands. Throughout the entire experiment, mice were housed in pairs in digital ventilated cages (DVC; Tecniplast SPA, Buguggiate (VA) Italy) with a relative humidity of 50–60%, temperature of 21 °C, light cycle of 7 a.m.–7 p.m., and ad libitum access to food and autoclaved water. During 6 weeks of baseline measurements, all mice were fed a conventional low-fat chow diet (chow, Sniff R/M-H diet V1534-703, Sniff Spezialdiäten GmbH, Soest, Germany). After baseline measurements, a young (4 months of age) baseline (chow) group of mice (*n* = 10) was sacrificed by transcardial perfusion to serve as a reference group. The remaining mice were equally divided into three groups (*n* = 16) that were matched for body weight, blood glucose, plasma cholesterol, and plasma triglyceride levels prior to treatment. Group 1 did not exercise and received a high-fat diet (HFD; D12451 Research Diets, New Brunswick, NJ, USA, 46.0% kcal fat, 36.0% carbohydrate and 18.0% kcal protein) for 6 months (HFD group, *n* = 16). A blocked running wheel (GYM500 activity wheel, Tecniplast S.p.A., Buguggiate (VA), Italy) was installed in their home cage as a control. Group 2 received the same HFD and had unlimited access to a functional running wheel in their home cage 24/7 for 6 months (HFD + exercise, *n* = 16). Group 3 was fed a BCAA-enriched HFD (HFD + BCAA, Research Diets, New Brunswick, NJ, USA, D12451 +0.75% valine, +0.75% isoleucine, +1.5% leucine) and had 24/7 access to a functional running wheel in their home cage for 6 months (HFD + BCAA + exercise, *n* = 16). After group allocation, mice remained housed in the same pairs as at baseline. The total casein content of the HFD + BCAA diet was reduced to ensure that the total protein intake was comparable to the standard HFD. A detailed composition of the HFD (+BCAA) is provided in Supplementary Table S1. Notably, in total, 4 animals of the HFD + BCAA + exercise group were excluded from analysis because they did not use the running wheel throughout the experiment. In addition, 2 mice died before the end of the experiment. The final group sample size was as follows: baseline (chow) *n* = 10, HFD *n* = 15, HFD + exercise *n* = 16, HFD + BCAA + exercise *n* = 11.

**Figure 1:**
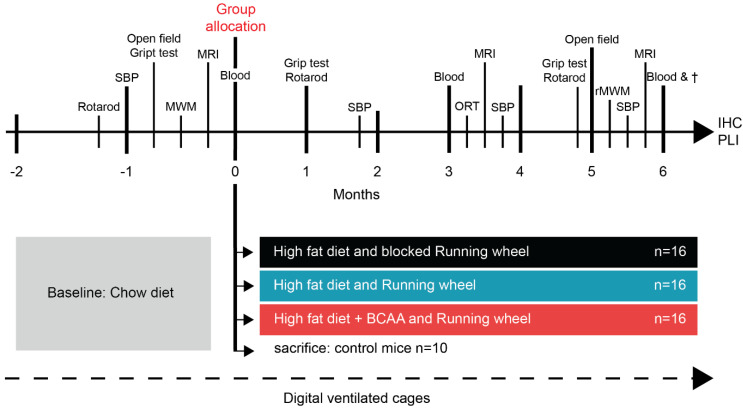
Study design. At 2 months of age (t = −2), 58 male Ldlr−/−.mice were selected for the present study and housed in pairs in digital ventilated cages. All mice were maintained on a standard chow diet during 8 weeks of baseline measurements. Baseline measurements included several experimental procedures, including systolic blood pressure (SBP) measurements, open field test, grip strength test, Morris water maze (MWM), and magnetic resonance imaging (MRI). Mouse pairs (sharing one home cage) were equally matched on the basis of body weight, fasting blood glucose, and plasma triglyceride and cholesterol levels (t = 0). (1) Group 1 (*n* = 16): a high-fat diet (HFD)-fed group that did not exercise and had a blocked running wheel in the cage. (2) Group 2 (*n* = 16): an exercising HFD-fed group (HFD + exercise) that had 24/7 access to a functional running wheel. (3) Group 3 (*n* = 16): an exercising HFD-fed group that received additional branched-chain amino acid (BCAA) supplementation and unlimited access to a functional running wheel. At 4 months of age (t = 0), another group of mice (*n* = 10) was sacrificed immediately after baseline measurements and organs (brain, adipose tissue, and muscle tissue) were used as healthy controls for post mortem analysis. After group allocation, mice were followed longitudinally up for 6 months, and behavioral tests and neuroimaging were repeated. Additionally, an object recognition test (ORT) and a reverse Morris water maze (rMWM) were performed. After 6 months of treatment (t = 6), animals were sacrificed by transcardial perfusion († = sacrifice), and immunohistochemical (IHC) analysis and polarized light imaging (PLI) were performed.

In this study, body weight was measured monthly. Food intake was measured weekly at cage level and normalized for the number of animals per cage (2 mice) to obtain the average individual food consumption. One mouse was excluded from the body weight analysis because it was identified as a statistical outlier at multiple time points (Supplementary Tables S4 and S5). All animals underwent physiological assessments (systolic blood pressure measurements and blood sampling), behavioral tests (open field test, grip strength test, rotarod, Morris water maze, and novel object recognition test), and MRI experiments were performed before, and longitudinally after, group allocation. At the end of the experiment, brains were harvested and white adipose tissue depots, including subcutaneous white adipose tissue (sWAT), epididymal white adipose tissue (eWAT), and mesenteric white adipose tissue (mWAT), together with hindlimb muscles (gastrocnemius, quadriceps, and soleus) were dissected and carefully weighed. Data on the weight of the fat depot are missing for three animals, and one mouse was considered to be a statistical outlier and was excluded from the analysis of muscle mass (Supplementary Tables S4 and S5). A detailed study design is shown in Figure 1.

### 2.2. Plasma Analysis

Blood samples were collected once just before group allocation (at baseline chow) and at three time points after group allocation (2, 3, and 6 months). After a fasting period of 5 h (8 a.m.–1 p.m.), blood was collected via a small tail cut. Blood glucose was measured immediately during blood sampling using a glucometer (FreeStyle Freedom Lite; Abbott Diabetes Care, Hoofddorp, The Netherlands). Plasma analysis was performed according to standardized protocols and assays [45]. Plasma cholesterol and triglycerides were measured with enzymatic assays (CHOD-PAP and GPO-PAP respectively; Roche Diagnostics, Almere, The Netherlands). Plasma insulin, E-selectin, and serum amyloid A (SAA) were measured using standardized ELISA kits (#90080; Crystal Chem, Elk Grove Village, IL, USA for insulin; R&D Systems, Inc., Minneapolis, MN, USA; #KMA0021; Thermo Fisher Scientific, Waltham, MA, USA for SAA). The final sample sizes varied by marker due to missing data and statistical outliers (Supplementary Tables S4 and S5).

### 2.3. Systolic Blood Pressure

Systolic blood pressure (SBP) was measured using a warmed tail-cuff plethysmography device (IITC Life Scientific Instruments, Woodland Hill, CA, USA) at several time points during the experiment (baseline and treatment months 2, 4, and 6) [41,42,55]. One habituation trial (10 measurements per mouse) was conducted at the beginning of the experiment. Afterwards, at each time point, all mice underwent 2 trials (each 10 measurements), with an intertrial interval of at least 1 h. The average SBP (millimeters of mercury, mmHg) of both trials (20 measurements) was calculated, excluding the first 3 measurements per trial (habituation) as well as invalid measurements (e.g., movement artifacts).

### 2.4. Home-Cage Activity

In this study, the home-cage activity of pair-housed mice was recorded 24/7 in digital ventilated cages (DVC) (Tecniplast S.p.A., Buguggiate (VA), Italy) throughout the experiment [56]. All system-specific details have been described previously [57]. Briefly, activity was monitored by a sensing board with 12 capacitive-based electrodes located underneath each DVC [57]. Every 250 ms, a proximity sensor measured the electrical capacitance of all electrodes. The electrical capacitance of the electrodes in proximity to a moving animal changes due to the dielectric properties of matter. As a result, mice moving over the electrodes were detected and recorded by a change in capacitance over a limited time interval. Activity was calculated as the absolute value of the difference between two consecutive measurements for each electrode. For the final analysis of relative cage activity, the electrode under the blocked and functional running wheel (electrode 6) was excluded, and we calculated the average activity of 11 electrodes per 12 h day and night. Only weekends were considered because of disruptions during the week (e.g., experiments and cage cleaning), and home-cage activity was divided by the number of mice per cage (*n* = 2) to express home-cage activity per mouse.

### 2.5. Running Wheel

After baseline measurements, running wheels were installed in all cages. The running wheels in the cages of the non-exercising HFD group were permanently blocked. The remaining two experimental groups (HFD + exercise and HFD + BCAA + exercise) had 24/7 access to functional running wheels. Total running distance (in kilometers) of pair-housed mice per cage was calculated per 12 h day and night. Only weekends were considered because of disruptions during the week (e.g., experiments and cage cleaning). Due to technical problems, 4 cages did not record the running distance during the last month of the experiment, and these cages were excluded from the analysis (Supplementary Tables S4 and S5).

### 2.6. Open Field Test

The open field test was used to assess locomotion, spontaneous exploratory behavior, and anxiety-related behavior. The open field test was performed twice: once at baseline and at 5 months after group allocation. Mice were individually placed in the center of a 45 × 45 × 30 cm box with transparent walls, where they had the chance to freely explore the arena for 10 min. All trials were videotaped with a camera attached to the ceiling above the open field. Walking distance, velocity, time spent in different zones (center, corners, and periphery), and the frequency of entering these zones were automatically calculated using EthoVisionXT 16 (Noldus Information Technology, Wageningen, The Netherlands). One mouse was identified as an outlier because it was inactive in the open field (Supplementary Tables S4 and S5).

### 2.7. Grip Strength Test

The grip strength test was performed to study muscle strength before and longitudinally after HFD (+BCAA) feeding and voluntary exercise. A grip strength meter (Grip Strength Meter, 47200, Ugo Basile, Gemonio, Italy) was used to measure both forelimb grip strength (2 paws) and fore- and hindlimb strength (4 paws) during 5 consecutive trials.

Forelimb grip strength was measured with the trapeze that was connected to the grip strength meter. Mice were held by the tail base and positioned over the trapeze, where they were allowed to grasp the bar. The mouse’s tail was gently pulled back until the mouse released the trapeze. At least 1 h later, fore- and hindlimb grip strength was measured by allowing the mice to grasp a grid with all 4 paws.

The grip strength meter automatically recorded the applied force every 0.05 s until the mouse released the grip. The recorded values were read out with the Data Collection Application 3 software (version 1.2.1.0, Ugo Basile, Gemonio, Italy), and different measures were analyzed: (1) the cumulative force, calculated as integral of force with respect to time of a single grip strength trial; and (2) the peak grip strength, which describes the highest recorded value per trial [58]. For each session, cumulative force (in gf) and average peak force (in gf) were calculated, excluding the first measurement (habituation) and invalid measurements. 

### 2.8. Rotarod

The rotarod (ITC LifeScience Inc., Woodland Hills, CA, USA) is a simple behavioral test of gross motor function that assesses an animal’s ability to maintain balance on a rod that is accelerating in speed. The initial speed was 4 rpm and increased to 40 rpm after 300 s. One habituation measurement per mouse was conducted at the beginning of the experiment. Afterwards, each mouse was tested 4 times with at least 1 h rest in between the measurements. The latency to fall from the rod was averaged per experimental group and is indicative of balance and coordination. Throughout the entire treatment period, the latency to fall decreased in all mice, indicating a worsening of motor coordination. No treatment differences were found (Supplementary Figure S1).

### 2.9. Novel Object Recognition Test

The novel object recognition test (ORT) is a commonly used behavioral test to assess various aspects of short-term memory in rodents, particularly recognition memory and novelty preference [48,59]. The experiment is described in detail in the Supplementary Materials and Methods. No treatment effects were observed (Supplementary Figure S11).

### 2.10. Morris Water Maze

Spatial learning and memory were tested using the Morris water maze (MWM) test. A circular white pool (108 cm diameter) was filled with 21 °C water made opaque with milk powder. A circular platform (8 cm diameter) was submerged 1 cm below the water surface in the center of the north-east quadrant. Four distal spatial cues were attached to the walls around the maze. The acquisition phase was conducted over 4 days, with 4 trials per day (1 h inter-trial interval), in which the animals were trained to find the hidden platform within 120 s. In each trial, the mice were placed in the pool from different cardinal points (south, north, west, and east). Animals that did not locate the platform within 120 s were manually placed on the platform for 30 s. The trial automatically stopped if the mice found the platform independently within 120 s and remained on the platform for at least 30 s. On day 4, a probe trial was performed, in which the platform was removed from the pool. The time animals spent in the former platform quadrant is indicative of spatial memory. 

#### Reverse Trial

A reverse MWM was used to assess memory retrieval after 6 months of treatment. A probe trial was performed to assess whether the mice remembered the former platform location (north-east, NE). Afterwards, the platform position was rotated 180° (south-west, SW) and the animals learned the new platform location within 2 days of acquisition, containing 4 trials per day, similar to the standard MWM session. On the second day, an additional probe trial was performed to measure spatial learning of the new platform location. 

All trials were videotaped with a ceiling camera to assess the escape latency (s), total swim distance (cm), and swim velocity. Additionally, for the probe trials, the cumulative time animals spent in the former platform location or platform quadrant was calculated (s) as well as the crossing of the former platform location or platform quadrant (frequency). EthoVision XT16 (Noldus, Wageningen, The Netherlands) was used to analyze all aforementioned read-outs automatically. In the first probe of the rMWM, one mouse was excluded from analysis due to its floating behavior in the water. In the second probe of the rMWM, the data of one mouse are missing since we have no video recording available due to technical problems (Supplementary Tables S4 and S5).

### 2.11. MRI Protocol: Cortical Thickness, Hippocampal Volume, ASL, DTI, and rsfMRI

Magnetic resonance imaging (MRI) measurements were performed on an 11.7T BioSpec Avance III small animal MR system (Bruker Biospin, Ettlingen, Germany) equipped with an actively shielded gradient set of 600 mT/m and operating on Paravision 6.0.1 software (Bruker, Karlsruhe, Germany). 

Before scanning, mice were fully anesthetized with isoflurane (induction: 3.5%, maintenance: 1.8%; Abbott Animal Health, Abbot Park, IL, USA) in a medical air and oxygen mixture (2:1). The head of the mouse was placed in a stereotactic holder to prevent motion artifacts during the scanning. Respiration was monitored with a pneumatic cushion respiratory monitoring system (Small Animal Instruments Inc., Stony Brook, New York, NY, USA). Body temperature, monitored with a rectal thermometer, was maintained at 37 °C with the help of a water heating pad. 

#### 2.11.1. Hippocampal Volume and Cortical Thickness

Hippocampal volume and cortical thickness were analyzed in ImageJ (ImageJ 1.51, National Institutes of Health, United States), using coronal T2-weighted images that were acquired at baseline, and after 3.5 and 6 months of treatment. Cortical thickness was measured manually in four different cortical areas that were selected, according to the mouse brain atlas of Franklin and Paxinos [60,61], at different Bregma levels: motor cortex (1.10), somatosensory cortex (−0.94), auditory cortex (−2.46), and visual cortex (−2.46) in the right and left hemispheres, respectively. The average cortical thickness of all measurements was calculated. Hippocampal volume was manually segmented on 7 consecutive coronal T2-weighted image slices covering the entire hippocampus. The boundaries of the hippocampus were set between Bregma −0.94 and −3.40, according to the mouse brain atlas of Franklin and Paxinos [60,61]. After segmentation, the hippocampal volume was calculated based on the sum of the segmented hippocampal surface area multiplied by the slice thickness. One mouse was excluded from analysis due to scanning artifacts (Supplementary Tables S4 and S5). Cortical thickness decreased after 3.5 months of treatment and remained below baseline levels after 6 months of treatment (Supplementary Figure S2). Notably, cortical thickness increased in HFD-fed mice between 3.5 and 6 months of treatment (Supplementary Figure S2). No changes in hippocampal volumetry were detected over time or between experimental groups (Supplementary Figure S2).

#### 2.11.2. Resting-State fMRI

Resting-state fMRI (rsfMRI) acquisition and analysis were performed to assess functional connectivity within specific brain regions, following the protocol of Zerbi et al. [62]. Eight animals were excluded from the analysis due to their low cerebral blood flow, which could potentially affect the results. In addition, 5 animals were identified as statistical outliers in the correlation analysis between different regions of interest (ROI) and were also excluded from the data set (Supplementary Tables S4 and S5).

#### 2.11.3. Cerebral Blood Flow

Cerebral blood flow (CBF) was measured via an established arterial spin labeling (ASL) method with a flow-sensitive alternating inversion recovery (FAIR) technique in different ROI (cortex, hippocampus, and thalamus), as previously described [62,63]. CBF measurements were performed under vasodilative conditions (medical air and oxygen mixture (2:1)) and vasoconstrictive conditions (pure oxygen). Subsequently, the cerebral vasoreactivity, which reflects the ability of blood vessels to adapt from a normal condition to a vasoconstrictive condition, was calculated using the following equation: Vasoreactivity = (CBF_vasoconstriction_ − CBF_vasodilation_)/CBF_vasodilation_. No treatment effects on the vasoreactivity were found (Supplementary Figure S3). Eight animals were identified as outliers due to low regional cerebral blood flow resulting from low body temperature under anesthesia (Supplementary Tables S4 and S5).

#### 2.11.4. Diffusion Tensor Imaging

GM and WM integrity were assessed using diffusion tensor imaging, as previously described [63,64]. Fractional anisotropy (FA) and mean water diffusivity (MD) were calculated, as reported elsewhere [65]. FA is an estimate of myelination and fiber density in the WM, while MD is an inverse measure of membrane density that is sensitive to GM changes [66,67,68]. FA and MD were measured in the overall WM and GM of the brain and, additionally, in manually selected ROI based on an anatomical atlas [60]. Two mice were excluded from the data set due to incorrect realignment of the scan, while five mice were excluded due to motion or EPI artifacts. Additionally, one mouse was excluded because it was a repeated statistical outlier in different ROI (Supplementary Tables S4 and S5). All imaging parameters are summarized in Supplementary Table S2. 

### 2.12. Tissue Preparation

Whole brains were extracted after transcardial perfusion with phosphate-buffered saline (PBS). The left hemisphere of each brain was post-fixed in 4% paraformaldehyde. After 24 h, the brains were transferred to 0.1 M PBS containing 0.01% sodium azide where the tissue was stored at 4 °C until sectioning. For cryoprotection, the brain hemispheres were placed in 30% sucrose in 0.1 M phosphate buffer overnight before sectioning into 30 μm free-floating coronal sections using a sliding microtome (Microm HC 440, Walldorf, Germany). A total of eight series were obtained and used for immunohistochemical stainings and polarized light imaging. 

### 2.13. Immunohistochemical Stainings

3,3′-diaminobenzidine-nickel (DAB-Ni) immunohistochemical stainings were performed according to a standardized protocol that was described previously [69]. The sections were pre-incubated (30 min) in blocking solution (3% BSA and 0.5% Triton X-100 in PBS) before incubation with primary antibodies. In total, three immunohistochemical stainings were performed. First, we stained for ionized calcium-binding adapter molecule (IBA-1), a commonly used marker specific for microglia/macrophages that is a measure of neuroinflammation. Second, we utilized doublecortin (DCX), a marker for premature neurons, as an indicator for neurogenesis. Third, we stained for glucose transporter 1 (GLUT-1), which is expressed in the cerebral microvasculature and reflects the microvascular integrity and quality. To this end, a series of free-floating sections were incubated with (1) polyclonal goat anti-IBA-1 (1:4000, Abcam, Cambridge, UK, Europe, RRID: AB_2340397), (2) polyclonal rabbit anti-DCX (1:4000, Synaptic Systems, Göttingen, Germany, Europe, RRID: AB_2620067), or (3) polyclonal rabbit anti-GLUT-1 (1:40,000, Millipore, Burlington, MA, USA, RRID: AB_11212210), respectively. As a secondary antibody, either donkey anti-goat biotin (1:1500, Jackson Immunoresearch, Cambridgeshire, UK, Europe AB_2340397) or donkey anti-rabbit biotin (1:1500, Thermo Scientific, Waltham, MA, USA, AB_228212) was used. Supplementary Figure S4 shows a representative image of the negative control for the GLUT-1 staining compared with a stained brain section. Stained sections were mounted on gelatin-coated slides. 

### 2.14. Quantification

Slides of all immunohistochemical stainings were scanned using a digital slide scanner (Aperio AT2, Leica Biosystems, Amsterdam, The Netherlands). Depending on the availability of brain tissue, up to three brain sections proximal to bregma 0.5 and −1.94 were selected, according to the atlas of Franklin and Paxinos [60,61]. ROI (bregma 0.5: cortex, corpus callosum, external capsule, basal ganglia, and anterior commissure; bregma −1.94: cortex, corpus callosum, external capsule, fimbria, hippocampus, internal capsule, optic tract, and thalamus) were selected in ImageJ using the freehand tool in a double-blinded fashion by two researchers. The different ROI were defined for further analysis when agreement was met for both researchers. An intensity-based threshold was defined in ImageJ to separate the target staining from non-specific background staining. Sample size per ROI can differ dependent on the staining quality and tissue availability, as described in detail in Supplementary Tables S4 and S5.

DCX-positive cells were quantified in the dentate gyrus of the hippocampus (−1.94 mm posterior to bregma) in 1 to 3 consecutive brain sections per mouse. Stained brain regions were counted at 40× magnification by two independent observers. No differences in the amount of DCX-positive cells were observed between treatment groups (Supplementary Figure S5).

IBA-1 and GLUT-1 density measurements were defined as the relative IBA-1+ or GLUT-1+ area per ROI. In addition, the intensity of the GLUT-1 staining, representing the amount of GLUT-1+, was calculated by subtracting the mean particle intensity from 255. The intensity of GLUT-1 staining is indicative of the quality of the microvasculature [70]. Intensity was quantified using ImageJ in the same regions as described above.

### 2.15. Polarized Light Imaging

Polarized light imaging (PLI) was performed to estimate myelin orientation and myelin density by utilizing the birefringent properties of myelin [71,72]. 

A series of 30 µm-thick coronal sections from the left brain hemisphere were mounted with demi water on an uncoated glass slide and dried at 37 °C for 24 h. The sections were cover-slipped with polyvinylpyrrolidone mounting medium. After drying for at least 1 week, raw PLI images were acquired using an Axio HV microscope (Zeiss, Germany) equipped with an RGB camera (AxioCam ERc 5s, Zeiss, Germany), a rotating polarizer, a quarter wave plate, a stationary polarizer, and a white LED light source [71,72,73,74]. Polarized light is generated by passing light through a linear polarizer combined with the quarter wave plate, the axis of which is oriented at a 45° angle to the polarizer plane. Subsequently, the polarized light passes through the brain tissue, followed by another polarizer that captures the change in polarization induced by tissue birefringence. Nine sequential images were taken at rotation angles between 0 and 160° using a 1.0× magnifying objective, yielding in a resolution of 4 µm/pixel. Additionally, background images were made for each rotation angle to correct for illumination differences between imaging sessions [71,75]. 

Post-processing of the raw images was performed with Matlab R2018b (MATLAB R2018b; MathWorks Inc., Natick, MA, USA). After background correction, different parameter maps were derived by fitting the PLI images to the Jones formula [71,76]. As a result, the following PLI maps were obtained: (1) transmittance, (2) retardance, (3) in-plane, (4) inclination, (5) dispersion, and (6) FOM-HSV map. 

Myelin density (retardance) and orientation (dispersion) were quantified with ImageJ. One brain section located proximal to bregma 0.50, 0.14, and/or −1.94 was selected, based on the atlas of Franklin and Paxinos [60]. In the retardance and dispersion maps, the ROI (bregma 0.5: motor cortex, somatosensory cortex, corpus callosum, external capsule, basal ganglia, and anterior commissure; bregma 0.14: anterior commissure, basal ganglia, corpus callosum, external capsule, motor cortex, somatosensory cortex, and fornix; and bregma −1.94: cortex, corpus callosum, external capsule, fimbria, hippocampus, internal capsule, and optic tract) were manually selected using the freehand tool in ImageJ by two researcher in a double-blinded manner (Supplementary Table S3). The different ROI were defined for further analysis when agreement was met for both researchers. Sample size per ROI varied according to tissue availability (e.g., damage in particular ROI) (Supplementary Tables S4 and S5). For both retardance and dispersion, the mean grey values are presented as percentages. 

### 2.16. Statistics

Data were analyzed in a statistical program (IBM SPSS Statistics 27, IBM Corporation, Armonk, NY, USA) and results are expressed as means ± standard error of the mean (SEM). 

First, all data sets were checked for outliers and the normality of the data distribution was tested using the Shapiro–Wilk test. The sample size per experiment is reported in Supplementary Table S4. Excluded outliers per experiment and the underlying reason for exclusion are described in Supplementary Table S5. If data were not normally distributed, they were transformed according to the Tukey Ladder of Powers. 

Second, we used a univariate general linear model to verify that data assessed at baseline did not show differences between the groups to which they were assigned later in the experiment. This ensured that group effects over time were not driven by baseline differences. If baseline differences were present, we corrected all data by dividing each time point by the corresponding baseline value. 

Third, all parameters that were assessed at multiple time points (body weight, caloric intake, blood plasma markers, MRI parameters, DVC data, Morris water maze, grip strength test, open field test, rotarod, and SBP) were analyzed using repeated measures ANOVA with a Bonferroni correction for multiple testing (‘group’ was selected as a between-subject factor). If overall group differences appeared to be significant (test of between-subject effect), a post hoc Tukey test (equal variance) or a Dunnett’s T3 (non-equal variance) was conducted to determine significant differences between the groups. Moreover, if a significant time by group interaction was found, the data set was split and the repeated-measures ANOVA was repeated to test for group-specific changes over time, and a univariate general linear model was performed to detect group differences at each of the time points used in the previous analysis. 

Finally, the remaining data were analyzed using multivariate ANOVA with Bonferroni correction for multiple comparisons (normally distributed) followed by a post hoc Tukey test (equal variance) or a Dunnett’s T3 (non-equal variance) to determine group differences. When data were not normally distributed, a Kruskal–Wallis was performed to analyze group differences. 

All significant results are reported in Supplementary Tables S6–S18.

## 3. Results

### 3.1. Body Weight, Caloric Intake, Fat Depots, and Muscle Mass

All 2-month-old mice were fed a standard chow diet during 8 weeks of baseline measurements (t = −2 to t = 0). Thereafter (t = 0), mice were treated for 6 months with (1) a high-fat diet (HFD), (2) HFD and exercise (HFD + exercise), or (3) a branched-chain amino acid-enriched HFD and exercise (HFD + BCAA + exercise). Throughout the experiment, mice were housed in pairs in DVC (t = −2 to t = 6). 

**Figure 2:**
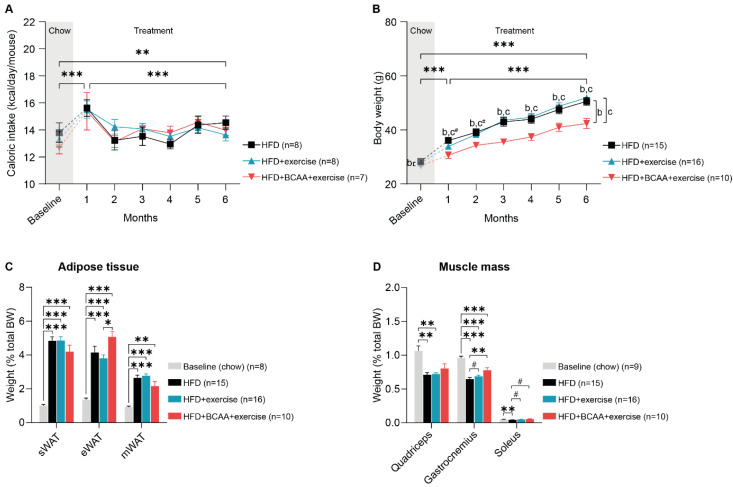
Body weight, caloric intake, fat depots, and muscle mass. The impact of exercise (HFD + exercise) and exercise combined with BCAA supplementation (HFD + BCAA + exercise) on (**A**) body weight, (**B**) caloric intake, (**C**) white adipose tissue depots (subcutaneous adipose tissue (sWAT), epididymal white adipose tissue (eWAT), and mesenteric white adipose tissue (mWAT)), and (**D**) muscle weight (quadriceps, gastrocnemius, and soleus) expressed as a percentage of total body weight (BW). High-fat diet (HFD), branched-chain amino acids (BCAA). Data are presented as mean ± SEM. * *p* < 0.05, ** *p* < 0.01, *** *p* < 0.001, # 0.05 < *p* < 0.08 (trend); b: significant difference HFD vs. HFD + BCAA + exercise group; c: significant difference HFD + exercise vs. HFD + BCAA + exercise group.

In the first month after the diet switch, caloric intake increased overall (Figure 2A). During 6 months of treatment, caloric intake decreased in all groups (Figure 2A). Six months after the diet switch, all animals still consumed more calories than when they were on chow diet (Figure 2A). Animals that received BCAA-enriched HFD consumed on average 3.22 g (±0.52 g) food per day throughout the 6-month study period, containing approximately 0.05 g (±0.01 g) isoleucine, 0.11 g (±0.02 g) leucine, and 0.06 g (±0.01 g) valine.

Already after 1 month of HFD and HFD + BCAA feeding, body weight increased significantly in all mice, with HFD + BCAA + exercise animals gaining significantly less weight than HFD-fed animals (Figure 2B). In the following treatment months, body mass continued to increase in all mice (Figure 2B). However, the body weight of the HFD + BCAA + exercise animals was significantly lower than that of both the HFD and HFD + exercise mice (Figure 2B). Throughout the 6-month treatment period, the HFD + BCAA + exercise mice weighed less than the HFD-fed animals, while the body weight became significantly different between HFD + BCAA + exercise and HFD + exercise animals starting at treatment month 3 (Figure 2B). When comparing the last treatment month with baseline measurements, all treatment groups significantly increased in weight, but the body weight of the HFD + BCAA + exercise animals was significantly lower than that of the HFD and HFD + exercise mice (Figure 2B).

At the end of the experiment, we dissected and carefully weighed the white adipose tissue depots, including subcutaneous white adipose tissue (sWAT), epididymal white adipose tissue (eWAT), and mesenteric white adipose tissue (mWAT), as well as the hind leg muscles (gastrocnemius, quadriceps, and soleus). All HFD-fed mice developed adiposity in different fat depots (Supplementary Figure S6A). While no treatment differences in WAT weights were observed (Supplementary Figure S6A), we found that eWAT mass as a proportion of total body mass was significantly higher in the HFD + BCAA + exercise mice compared with the HFD + exercise mice (Figure 2C).

As a result of increased body fat percentage in all HFD-fed mice (Supplementary Figure S6B), the percentages of quadriceps, gastrocnemius, and soleus muscle mass were significantly lower in the HFD group compared with the young chow-fed mice (Figure 2D). Similarly, the relative weight of the quadriceps was decreased in the HFD + exercise mice, and the percentage of gastrocnemius mass was lower in the HFD + exercise and HFD + BCAA + exercise animals than in the young chow-fed mice (Figure 2D). In contrast, exercise and BCAA prevented the decrease in relative gastrocnemius and soleus muscle mass. Likewise, the relative soleus muscle mass was similar between the HFD + exercise and young chow-fed animals (Figure 2D). Six months of exercise slightly increased the proportion of gastrocnemius and soleus mass compared with non-exercising mice (Figure 2D). Supplementation with BCAA resulted in a significantly increased proportion of gastrocnemius mass and a slightly increased proportion of soleus muscle mass (Figure 2D). Importantly, absolute differences in muscle mass were only observed in HFD + exercise vs HFD animals (Supplementary Figure S6C), indicating that the beneficial effects of BCAA and exercise on muscle percentage are related to reduced body weight. 

### 3.2. Metabolic Factors and Systolic Blood Pressure

**Figure 3:**
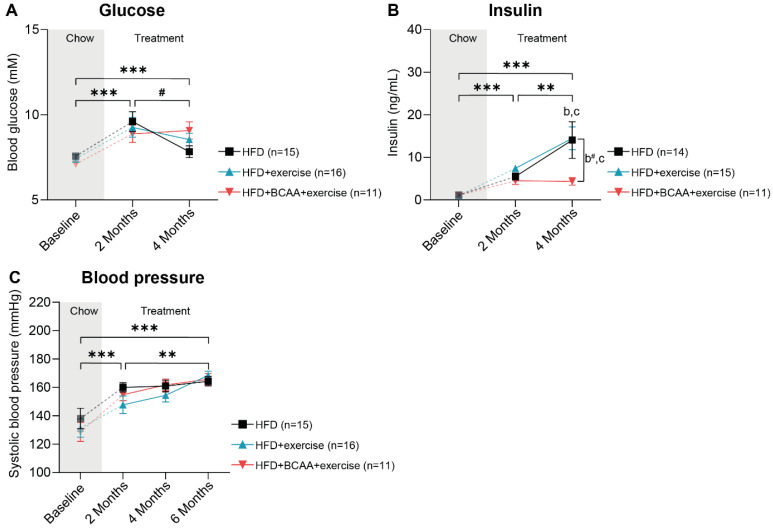
Metabolic markers and systolic blood pressure. (**A**) Fasting blood glucose levels and (**B**) plasma insulin levels measured in the HFD, HFD + exercise and HFD + BCAA + exercise groups at baseline (chow) and after 3 and 6 months of treatment. (**C**) Systolic blood pressure was assessed in mice of all experimental groups at baseline and at three separate time points after group allocation (2, 4, 6 months). High-fat diet (HFD), branched-chain amino acids (BCAA). Data are presented as mean ± SEM. ** *p* < 0.01, *** *p* < 0.001, # 0.05 < *p* < 0.08 (trend); b: significant difference HFD vs. HFD + BCAA + exercise group, c: significant difference HFD + exercise vs. HFD + BCAA + exercise group.

We assessed the impact of exercise and the synergistic effect of exercise and BCAA supplementation on obesity-related metabolic risk factors longitudinally. After 3 months of treatment, fasting blood glucose levels doubled in all experimental groups compared with the baseline (Figure 3A). Although blood glucose levels decreased slightly between treatment months 3 and 6, fasting glucose remained significantly higher compared with the baseline (Figure 3A). Of note, mice receiving HFD + BCAA + exercise treatment had lower insulin levels between 3 and 6 months of treatment, as well as between baseline and 6 months, compared with mice that only exercised (Figure 3B). Additionally, between baseline and 6 months of treatment, insulin was slightly lower in the HFD + BCAA + exercise mice compared with the HFD mice (Figure 3B). This effect was mainly driven by significantly lower insulin levels in the HFD + BCAA + exercise animals compared with the HFD and HFD + exercise mice at 6 months of treatment (Figure 3B). Cholesterol, triglycerides, and serum amyloid A (SAA) levels increased throughout the entire treatment period, and no treatment effects were observed (Supplementary Figure S7A–C). Furthermore, after an initial increase at 2 months of treatment, E-selectin levels started to decrease, resulting in levels similar to the baseline after 6 months of treatment (Supplementary Figure S7D). Systolic blood pressure was assessed bimonthly and increased throughout the entire treatment period, with no differences between groups (Figure 3C). 

### 3.3. Voluntary Exercise, Home-Cage Activity, and Locomotive Behavior

#### 3.3.1. Home-Cage Activity

**Figure 4:**
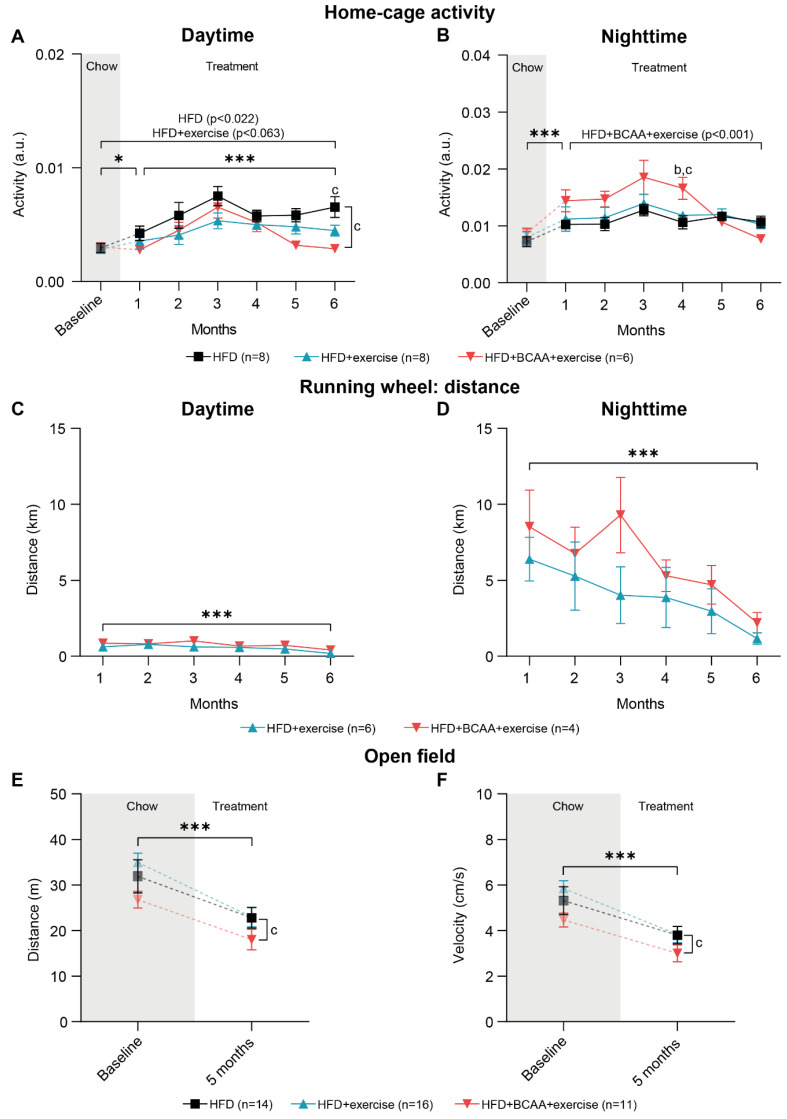
Home-cage activity, voluntary exercise, and open field test. Digital ventilated cages recorded home-cage activity and voluntary exercise of HFD, HFD + exercise, and HFD + BCAA + exercise mice 24/7 at baseline (chow) and during the consecutive 6 treatment months. In detail, average home-cage activity during (**A**) daytime and (**B**) nighttime as well as total running distance on the running wheel during (**C**) daytime and (**D**) nighttime were assessed. Moreover, the effect of exercise and exercise in combination with BCAA supplementation on (**E**) distance and (**F**) velocity during a 10-min exploration period were analyzed in an open field test at baseline and after 5 months of treatment. High-fat diet (HFD), branched-chain amino acids (BCAA). Data are presented as mean ± SEM. * *p* < 0.05, *** *p* < 0.001; b: significant difference HFD vs. HFD + BCAA + exercise group, c: significant difference HFD + exercise vs. HFD + BCAA + exercise group.

Home-cage activity of pair-housed mice was assessed 24/7 day and night (12 h interval) in DVC cages. Home-cage activity was significantly higher during the night than during the day (Figure 4A,B). One month after the diet switch and installation of the running wheels, home-cage activity increased in all groups during the day (Figure 4A) and night (Figure 4B). Throughout 6 months of treatment, daytime activity continued to increase in all mice; however, the HFD + BCAA + exercise animals were less active than mice on HFD (Figure 4A). Although overall daytime activity increased during the time of treatment, only HFD mice displayed higher daytime activity during the last month of the treatment when compared to baseline, and the HFD + exercise mice tended to be more active compared to baseline (Figure 4A). Furthermore, the HFD + BCAA + exercise mice were significantly less active than mice in the HFD group at treatment month 6 (daytime) (Figure 4A). 

In contrast to home-cage activity during the day, nighttime activity did not change in the HFD and HFD + exercise mice, while decreased nighttime activity was measured in the HFD + BCAA + exercise mice during the 6 months of treatment (Figure 4B). This effect was driven by the non-significantly higher nighttime activity of the HFD + BCAA + exercise mice compared with the other experimental groups at treatment months 1–3, as well as the significantly higher nighttime activity of the HFD + BCAA + exercise animals compared with the HFD and HFD + exercise mice at 4 months of treatment, followed by decreasing nighttime activity in the home cage after treatment month 4 (Figure 4B). After 6 months of treatment, nighttime activity was similar to baseline in all experimental groups (Figure 4B).

#### 3.3.2. Running Wheel Distance

As a measure of voluntary exercise, the total distance run on the running wheel inside the home cage was monitored day and night (12 h time interval) (Figure 4C,D). Mice ran significantly more at night than during the day (Figure 4C,D). During both daytime and nighttime, the running distance decreased over time in both exercise groups (Figure 4C,D).

#### 3.3.3. Open Field Test

In addition to advanced activity and exercise monitoring of pair-housed mice in the DVC, locomotor ability and anxiety-related behavior were individually tested in the open field at baseline and 5 months after group allocation. Both total walking distance and walking velocity decreased significantly over time in all experimental groups (Figure 4E,F). Notably, walking distance and velocity were significantly lower in the HFD + BCAA + exercise group compared to the HFD + exercise animals (Figure 4E,F). After 5 months of treatment, mice in all groups spent more time in the corners of the open field, indicating increased anxiety-related behavior (Supplementary Figure S8B). Additionally, the frequency of entering the corners and periphery of the open field decreased, suggesting reduced locomotor and exploratory behavior (Supplementary Figure S8E,F). Notably, the HFD + BCAA + exercise mice entered the corners and periphery less frequently compared to the HFD + exercise mice (Supplementary Figure S2E,F).

### 3.4. Muscle Strength

**Figure 5:**
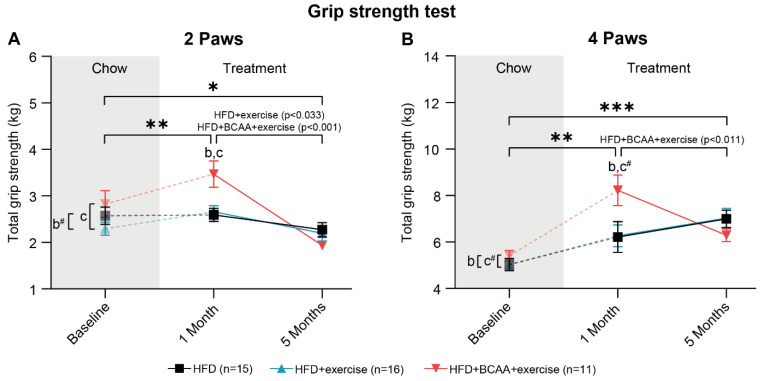
Grip strength test. Total muscle strength of (**A**) forelimbs (2 paws) and (**B**) fore- and hindlimbs combined (4 paws), was measured at baseline (chow) and after 1 and 5 months of treatment with exercise and exercise combined with BCAA supplementation. High-fat diet (HFD), branched-chain amino acids (BCAA). Data are presented as mean ± SEM. * *p* < 0.05, ** *p* < 0.01, *** *p* < 0.001, # 0.05 < *p* < 0.08 (trend); b: significant difference HFD vs. HFD + BCAA + exercise group, c: significant difference HFD + exercise vs. HFD + BCAA + exercise group.

The grip strength test was performed to test whether the combination of voluntary exercise and BCAA supplementation improves total strength (Figure 5A,B) and peak strength (Supplementary Figure S9) of forelimbs (two paws) and combined fore- and hindlimbs (four paws). One month after group allocation, all animals showed increased total forelimb muscle strength (Figure 5A). After the first month of treatment, the mice on HFD + BCAA + exercise exerted more forepaw force than both the HFD-fed mice (trend) and HFD + exercise animals (Figure 5A). Together, these data suggest that dietary BCAA supplementation transiently improved forelimb muscle strength. Between 1 and 5 months of treatment, both the HFD + exercise and HFD + BCAA + exercise mice lost forepaw strength, resulting in equal total forepaw strength among all experimental groups after 5 months of treatment (Figure 5A). Notably, all animals displayed lower forepaw strength after 5 months of treatment compared to baseline measurements (Figure 5A). 

In addition, total fore- and hindlimb grip strength (four paws) increased in all experimental groups between baseline and 1 month of treatment, with the HFD + BCAA + exercise mice being stronger than mice in the HFD group and tending to be stronger than the HFD + exercise mice (Figure 5B). Similar to forelimb strength, BCAA increased total four-paw grip strength compared to the other treatment groups after 1 month of treatment (Figure 5B). Moreover, four-paw grip strength decreased in the HFD + BCAA + exercise animals between treatment months 1 and 5, resulting in equal total strength among all experimental groups at the end of the experiment (Figure 5B). Four-paw grip strength was higher in all mice after 5 months of treatment compared to baseline measurements (Figure 5B).

### 3.5. Cognition: Morris Water Maze

#### 3.5.1. Morris Water Maze

**Figure 6:**
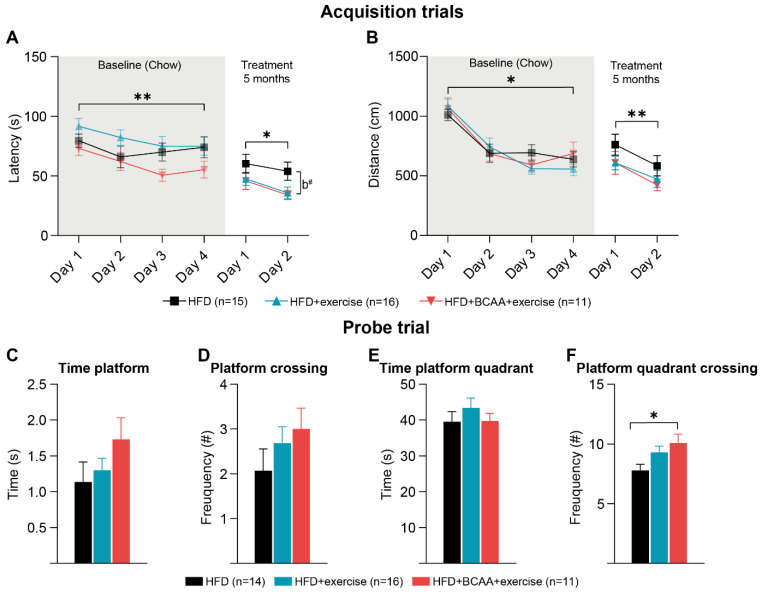
(Reverse) Morris water maze. Spatial learning and memory were assessed in the Morris water maze (MWM) at baseline (chow) and in a reverse MWM 5 months after allocation in treatment groups (HFD, HFD + exercise, HFD + BCAA + exercise). (**A**) During 4 constitutive acquisition days, chow-fed mice learned to find the hidden platform. After 5 treatment months, animals of all treatment groups were again subjected to an acquisition trial (2 days) with a novel platform location. The latency to find the hidden platform is indicative of spatial learning ability. (**B**) Total swim distance was measured during both acquisition phases (baseline and reverse). (**C**–**F**) In the probe trial of the reverse MWM, the platform was removed from the pool, and (**C**) the time spent at the former platform location, (**D**) frequency of crossing the former platform location, (**E**) time spent in the former platform quadrant, and (**F**) the frequency of crossing the former platform quadrant were measured. High-fat diet (HFD), branched-chain amino acids (BCAA). Data are presented as mean ± SEM. * *p* < 0.05, ** *p* < 0.01, # 0.05 < *p* < 0.08 (trend); b: difference HFD vs. HFD + BCAA + exercise group.

The MWM was performed to evaluate spatial learning and hippocampal-dependent memory. All young chow-fed mice demonstrated the ability to learn the location of the hidden platform in a 4-day acquisition trial, as reflected by the decreased time to find the hidden platform location (Figure 6A) and total swim distance (Figure 6B). No differences in spatial learning ability were found between groups at baseline (Figure 6A). In a baseline probe trial, hippocampal-dependent memory formation was similar in all mice. No differences were measured in the time and frequency that mice spent at the platform location and former platform quadrant (Supplementary Figure S10A–D).

#### 3.5.2. Reverse Morris Water Maze

After 5 treatment months, a reverse MWM was performed. First, the probe trial was repeated to evaluate the effect of HFD + exercise and HFD + BCAA + exercise on long-term memory. No significant differences were detected between groups in the time and frequency that mice spent in the former platform location and former platform quadrant (Supplementary Figure S10E–H). In addition, animals learned to find a novel platform location during a 2-day acquisition trial (Figure 6A,B). All animals were able to find the novel platform location faster on acquisition day 2 (Figure 6A), and their total swimming distance decreased significantly (Figure 6B). Notably, the HFD + BCAA + exercise group tended to find the hidden platform faster than the HFD mice (Figure 6A). Subsequently, mice were re-tested in a probe trial to assess their ability to remember the novel platform location (Figure 6C–F). While there were no group differences in platform crossing and time spent in the novel platform location (Figure 6C,D), mice of the HFD + BCAA + exercise group entered the platform quadrant more frequently than the HFD mice (Figure 6F). The time animals spent in the former platform quadrant did not change between experimental groups (Figure 6E). 

### 3.6. Functional Connectivity

**Figure 7:**
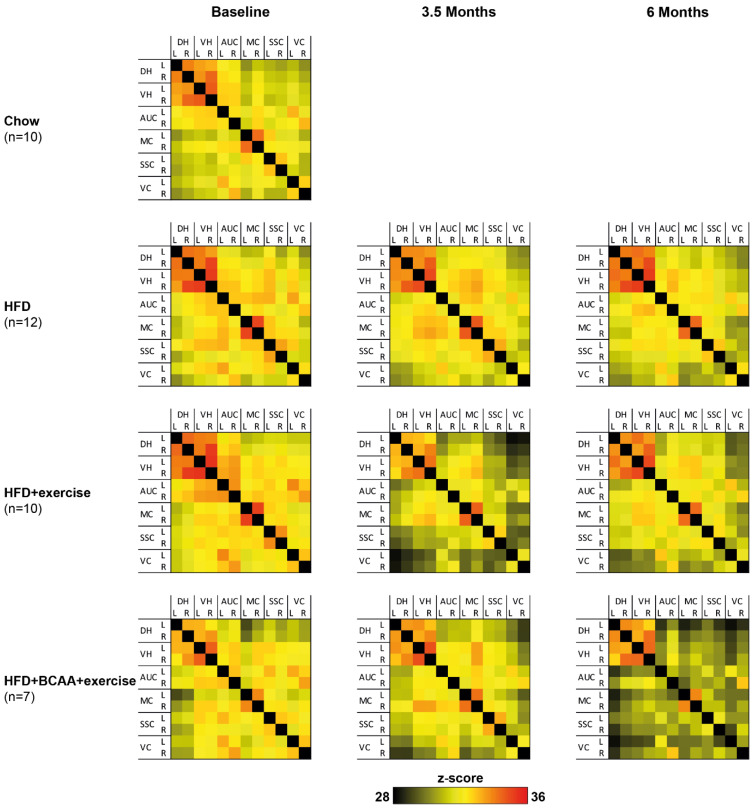
Resting-state functional connectivity based on total correlations. Functional connectivity between brain regions was measured using resting-state fMRI at baseline (chow) and after 3.5 and 6 months of treatment with HFD, HFD + exercise, and HFD + BCAA + exercise. Total correlation matrixes show the connectivity between the dorsal hippocampus (DH), ventral hippocampus (VH), auditory cortex (AC), motor cortex (MC), somatosensory cortex (SSC), and visual cortex (VC) for the left (L) and right (R) hemisphere, respectively, at each neuroimaging time point, respectively. High-fat diet (HFD), branched-chain amino acids (BCAA).

To determine the effect of exercise and dietary BCAA supplementation on functional connectivity patterns in the brain, resting-state fMRI data were statistically analyzed based on total correlation (Figure 7) and partial correlation (Supplementary Figure S12) for each neuroimaging time point, respectively. We analyzed intrahemispheric as well as interhemispheric connections between different cortical areas (auditory cortex, motor cortex, somatosensory cortex, and visual cortex) and hippocampal areas (dorsal hippocampus and ventral hippocampus). All significant results are summarized in Supplementary Tables S11 and S12. 

#### 3.6.1. Total Correlation

Between baseline and 3.5 months of treatment, intra- and interhemispheric connectivity decreased in multiple cortical and hippocampal regions of all experimental groups. Specifically, functional connectivity between visual cortex, somatosensory cortex, auditory cortex, and the other included ROI was affected. Likewise, functional connectivity of the auditory cortex and other ROI decreased over time. Reduced functional connectivity patterns were persistent, as similar effects were also detected after 6 months of treatment. Group differences were also measured between baseline and 6 months of treatment. BCAA decreased functional connectivity between the (1) left motor cortex and left dorsal hippocampus and (2) right ventral hippocampus and left dorsal hippocampus compared to the other treatment groups. 

#### 3.6.2. Partial Correlations

Partial correlation analysis emphasizes the direct connectivity between brain regions, while regressing the temporal BOLD signal from all other ROI. In contrast to the total correlation analysis, intra- and interhemispheric functional connectivity increased between different cortical and hippocampal areas, and increased between baseline and 3.5 months of treatment, between 3.5 and 6 months of treatment, and between baseline and 6 months of treatment, in all animals. Group-specific changes over time were mostly found intrahemispherically between cortical regions. 

Several treatment effects on functional connectivity were observed between baseline and 6 months of treatment. Exercising mice showed a decreased functional connectivity between the left somatosensory cortex and right motor cortex. Functional connectivity between the right ventral hippocampus and left dorsal hippocampus was decreased in BCAA-supplemented mice compared to HFD + exercise animals. In contrast, mice in the HFD + BCAA + exercise group showed higher functional connectivity between the left visual cortex and left dorsal hippocampus than exercising animals without BCAA supplementation.

### 3.7. Vascular Integrity and Quality

CBF was measured under vasodilative and vasoconstrictive conditions at baseline, 3.5 months, and 6 months after group allocation. To assess the ability of the vasculature to respond to vasoconstrictive stimuli, vasoreactivity was calculated. Impaired vasoreactivity is an indicator of vascular impairment. 

#### 3.7.1. CBF: Vasodilative Conditions

**Figure 8:**
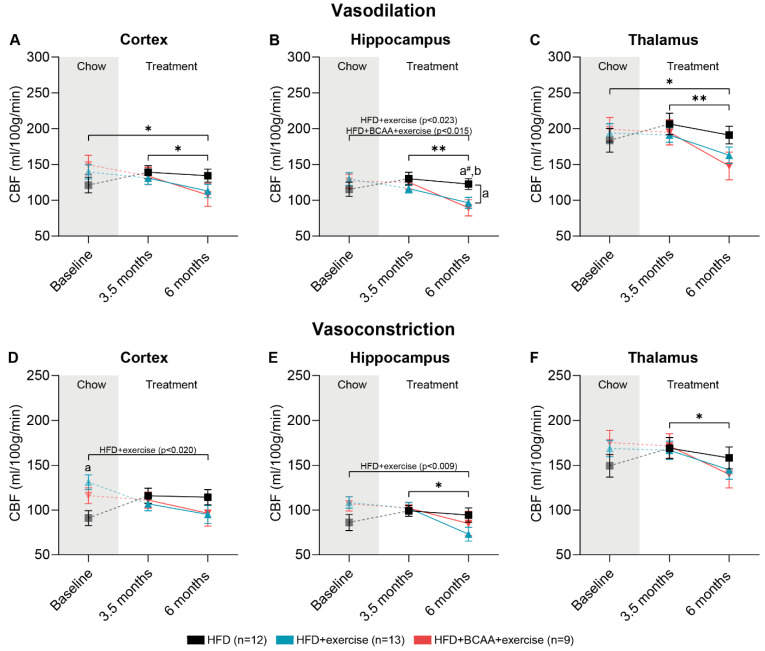
Measurement of cerebral blood flow under vasodilative and vasoconstrictive conditions. Cerebral blood flow (CBF) was measured by arterial spin labeling at baseline (chow) and after 3.5 and 6 months of treatment with HFD, HFD + exercise, and HFD + BCAA + exercise. At each neuroimaging session, CBF was first assessed under vasodilative conditions in the (**A**) cortex, (**B**) hippocampus, and (**C**) thalamus, and afterwards under vasoconstrictive conditions in the (**D**) cortex, (**E**) hippocampus, and (**F**) thalamus. High-fat diet (HFD), branched-chain amino acids (BCAA), cerebral blood flow (CBF). Data are presented as mean ± SEM. * *p* < 0.05, ** *p* < 0.01, # 0.05 < *p* < 0.08 (trend); a: significant difference HFD vs. HFD + exercise group, b: significant difference HFD vs. HFD + BCAA + exercise group.

After 3.5 months of treatment, there were no differences in CBF in the cortex, hippocampus, and thalamus compared to baseline measurements (Figure 8A–C). In all experimental groups, CBF decreased in the cortex, hippocampus, and thalamus (Figure 8A–C) between 3.5 and 6 months of treatment. During the same time period, the HFD + exercise mice had significantly lower hippocampal CBF compared with the HFD mice (Figure 8B). Notably, after 6 months of treatment, both exercise groups demonstrated decreased hippocampal CBF (Figure 8B). This was due to decreased hippocampal CBF in the HFD + BCAA + exercise and HFD + exercise groups between baseline and 6 months, whereas the HFD-fed mice did not show changes in hippocampal CBF (Figure 8B). Furthermore, CBF decreased in the cortex and thalamus of all experimental groups after 6 months of treatment compared to baseline measurements (Figure 8A,C). 

#### 3.7.2. CBF: Vasoconstrictive Conditions

After exposing mice to pure medical oxygen, healthy blood vessels should constrict, resulting in an overall lower CBF. Between baseline and 6 months of treatment, the HFD + BCAA + exercise mice tended to have lower cortical CBF than the HFD + exercise mice (Figure 8D). Moreover, CBF decreased in the hippocampus and thalamus of all animals between 3.5 months and 6 months of treatment (Figure 8E,F). When comparing CBF after 6 months of treatment with baseline measurements, HFD + exercise treatment resulted in decreased CBF in the cortex and hippocampus (Figure 8D,E). 

#### 3.7.3. Glucose Transporter-1

**Figure 9:**
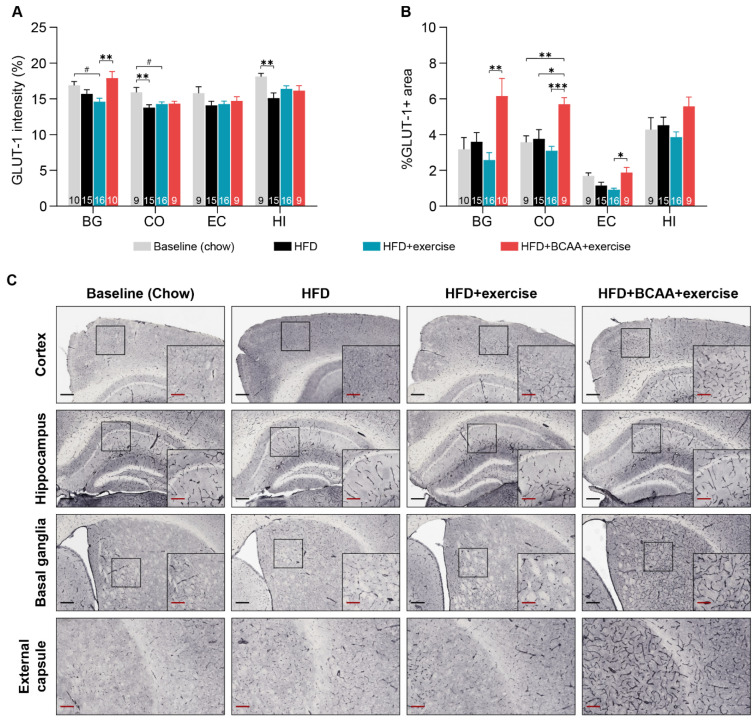
Cerebrovascular integrity. Immunohistochemical analysis of glucose transporter 1 (GLUT-1) as a measure of cerebrovascular integrity in different brain regions of interest (basal ganglia (BG), cortex (C), external capsule (EC), and hippocampus (HI)). (**A**) GLUT-1 staining intensity is a measure of the amount of GLUT-1 in the capillaries. (**B**) The percentage of GLUT-1 positive staining per area is representative of vascular density. (**C**) Representative images of the GLUT-1 staining (black scale bar = 200 µm; red scale bar = 100 µm). Group differences between young chow-fed animals (baseline), HFD, HFD + exercise, and HFD + BCAA + exercise groups were analyzed. High-fat diet (HFD), branched-chain amino acids (BCAA). Data are presented as mean ± SEM. * *p* < 0.05, ** *p* < 0.01, *** *p* < 0.001, # 0.05 < *p* < 0.08 (trend).

Glucose transporter-1 (GLUT-1) is specifically expressed in the cerebral microvasculature. The amount of GLUT-1 positive (GLUT-1+) staining was assessed by measuring the intensity of GLUT-1 immunohistochemical staining in different ROI (Figure 9A). In addition, we determined the relative area of GLUT-1+ staining as a measure of capillary density (Figure 9B). 

After 6 months of HFD feeding, the amount of GLUT-1+ in the cortex and hippocampus decreased compared to young chow-fed mice (Figure 9A). In contrast, in the other treatment groups, the amount of GLUT-1+ in the cortex and hippocampus was comparable to that in the young chow-fed mice, indicating a subtle preventive effect of exercise, and BCAA + exercise. Only exercising mice tended to display lower amounts of GLUT-1+ in the basal ganglia and cortex compared to young chow-fed animals (Figure 9A). Importantly, BCAA supplementation restored the amount of GLUT-1+ in the basal ganglia (Figure 9A). 

Cortical capillary density, measured in the HFD + BCAA + exercise mice, was significantly elevated in comparison with the HFD and HFD + exercise and young chow-fed mice, reflecting a possible BCAA effect (Figure 9B). Moreover, animals of the HFD + BCAA + exercise group displayed higher capillary density than the HFD + exercise animals in the basal ganglia and external capsule (Figure 9B). However, within the same regions, all treatment groups had a vascular density similar to that of young chow-fed mice (Figure 9B). 

### 3.8. Microstructural Changes in Grey and White Matter

#### 3.8.1. Diffusion Tensor Imaging

**Figure 10:**
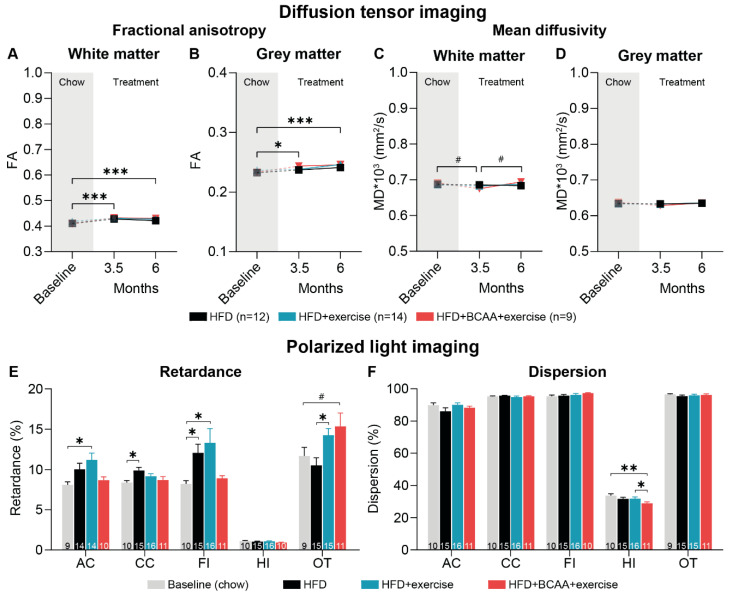
Grey matter and white matter integrity. Diffusion tensor imaging was performed in vivo to investigate grey and white matter integrity of HFD, HFD + exercise, and HFD + BCAA + exercise animals at baseline (chow) and after 3.5 and 6 months of treatment. Fractional anisotropy (FA) and mean diffusivity (MD) were assessed in (**A**,**C**) white matter and (**B**,**D**) grey matter. Additionally, polarized light imaging (PLI) was performed post mortem to quantify myelin density, based on the (**E**) retardance map, as well as fiber orientation, based on the (**F**) dispersion map, in different brain regions, including the anterior commissure (AC), corpus callosum (CC), fimbria (FI), hippocampus (HI), and optic tract (OT). Group differences between young chow-fed animals (baseline), HFD, HFD + exercise, and HFD + BCAA + exercise animals were analyzed. High-fat diet (HFD), branched-chain amino acids (BCAA). Data are presented as mean ± SEM. * *p* < 0.05, ** *p* < 0.01, *** *p* < 0.001, # 0.05 < *p* < 0.08 (trend).

GM and WM integrity was longitudinally assessed using in vivo diffusion tensor imaging (Figure 10). We analyzed fractional anisotropy (FA) as a measure of myelination and fiber density, and mean diffusivity (MD) as an inverse measure of membrane density. FA and MD were assessed in the total WM and GM (Figure 10A–D) as well as region specific (auditory cortex, basal ganglia, corpus callosum, fornix, hippocampus, motor cortex, optic tract, and somatosensory cortex) (Supplementary Table S15). 

Between baseline and 3.5 months after treatment, FA increased in the total WM and GM and region specific in the basal ganglia, corpus callosum, fornix, and the optic tract of all animals (Figure 10A,B and Supplementary Table S15). This effect remained significant after 6 months of treatment (Figure 10A,B). Interestingly, exercise, but not exercise in combination with BCAA, increased FA in the corpus callosum and fornix between baseline and 6 months of treatment in comparison with HFD mice (Supplementary Table S15). 

MD tended to decrease in the total WM and region specific in the basal ganglia, optic tract, and somatosensory cortex of all mice between baseline and 3.5 months of treatment (Figure 10C and Supplementary Table S15). Importantly, exercise and exercise in combined with BCAA supplementation resulted in decreased MD levels in the fornix after 3.5 months of treatment (Supplementary Table S15). After 3.5 months of treatment, MD was lower in the HFD + BCAA + exercise mice than in the HFD-fed mice (Supplementary Table S15). Together, the data indicate that exercise and BCAA improved tissue integrity in the fornix. From 3.5 months to 6 months of treatment, MD decreased in the fornix and corpus callosum of HFD-fed animals (Supplementary Table S15).

#### 3.8.2. Polarized Light Imaging

In addition to DTI, we performed polarized light imaging (PLI) to quantify myelin density and orientation in the brains post mortem. PLI retardance values are representative for myelination, whereby decreased retardance values can be used as an indirect measure of myelin loss. Myelin density was elevated in the corpus callosum and the fimbria of the HFD-fed animals compared to the young chow-fed mice (Figure 10E). In the anterior commissure, as well as in the fimbria, the HFD + exercise mice displayed more myelinated fibers than the young chow-fed animals (Figure 10A,E). In addition, animals in both the HFD + exercise and HFD + BCAA + exercise groups showed higher myelin density in the optic tract than the HFD-fed mice (Figure 10E). 

Dispersion levels are a quantitative estimate of fiber orientation. Lower dispersion values are a measure of better myelin quality. Notably, treatment with exercise and BCAA resulted in decreased dispersion in the hippocampus compared to the HFD + exercise mice and young chow animals (Figure 10F). 

### 3.9. Neuroinflammation

**Figure 11:**
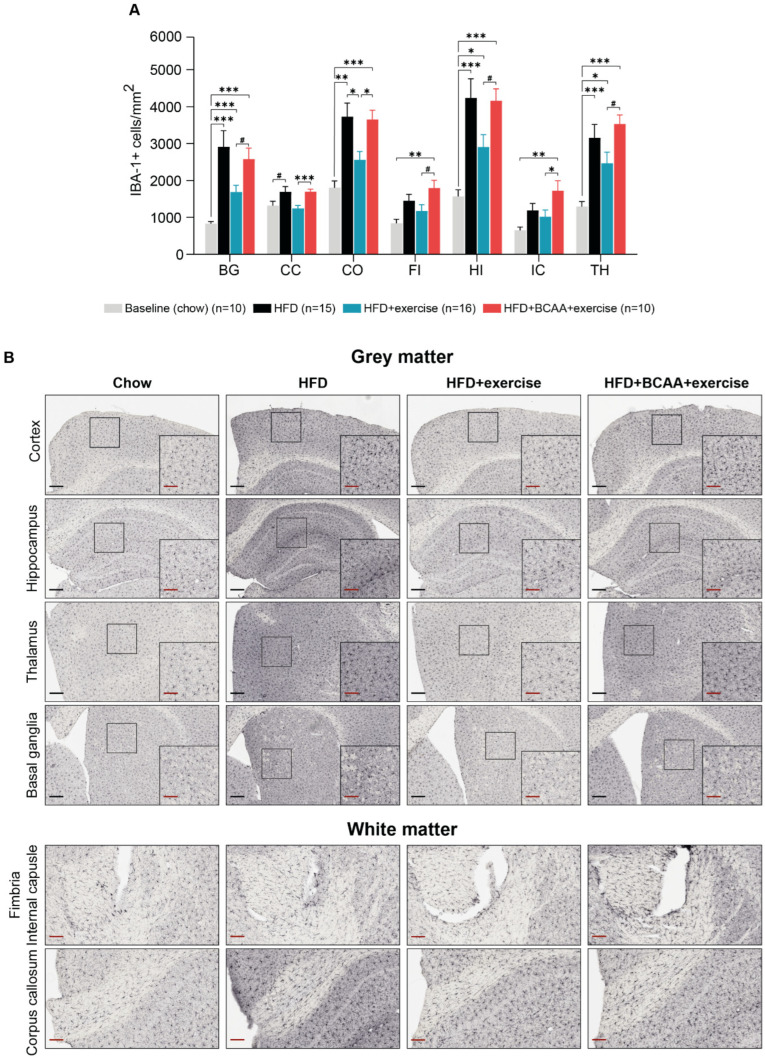
Neuroinflammation. Immunohistochemical analysis of ionized calcium-binding adapter molecule (IBA-1), as a measure of neuroinflammation in different brain regions of interest (basal ganglia (BG), corpus callosum (CC), cortex (C), fimbria (FI), hippocampus (HI), internal capsule (IC), and thalamus (TH)). (**A**) The number of IBA-1+ cells/mm^2^ was analyzed in young chow-fed animals (baseline), HFD, HFD + exercise, and HFD + BCAA + exercise groups. (**B**) Representative images of the IBA-1 staining (black scale bar = 200 µm; red scale bar = 100 µm). High-fat diet (HFD), branched-chain amino acids (BCAA). Data are presented as mean ± SEM. * *p* < 0.05, ** *p* < 0.01, *** *p* < 0.001, # 0.05.

Neuroinflammation was assessed by analyzing the count of IBA-1+ cells (Figure 11A) and the relative area of IBA-1+ staining (Supplementary Figure S13) in the brain. IBA-1 is specifically expressed in activated microglia and macrophages.

All HFD-fed mice had more activated microglia (IBA-1+ cells) in certain brain regions, such as the basal ganglia, hippocampus, and thalamus, compared to young mice that were fed a regular diet (chow) (Figure 11A). In the cortex, the number of activated microglia was also higher in the HFD-fed mice and in the HFD + BCAA + exercise mice than in the young chow-fed mice (Figure 11A). In contrast, mice that were treated with exercise alone did not show an increase in activated microglia in the cortex and corpus callosum compared to young chow-fed mice (Figure 11A). Interestingly, the HFD + BCAA + exercise mice had more activated microglia in the fimbria and internal capsule than the young chow-fed mice, while none of the other treatment groups showed increased activated microglia in the same ROI (Figure 11A). Similarly, the HFD + BCAA + exercise mice showed significantly more activated microglia than exercising mice not receiving BCAA supplementation in several regions of interest (ROI), including the basal ganglia, corpus callosum, cortex, internal capsule, hippocampus, anterior commissure, fimbria, and thalamus (Figure 11A).

## 4. Discussion

The objective of this study was to investigate the preventive effect of voluntary exercise and the possible synergistic effect of co-treatment of voluntary exercise with dietary supplementation of BCAA on obesity-related structural and functional brain changes, as well as subsequent behavioral alterations in Ldlr−/−.Leiden mice on an HFD. In this study, metabolic risk factors, including body weight, white adipose tissue accumulation, hypercholesterolemia, hypertriglyceridemia, hyperinsulinemia, and hypertension could not be attenuated by exercise alone. However, we demonstrated that exercise alone improved WM integrity, altered CBF, and attenuated neuroinflammation in the brain. To our knowledge, this is the first study that has investigated the synergistic effect of exercise and BCAA on brain structure and function. We found that exercising mice treated with dietary BCAA did not exhibit beneficial effects on neuroimaging parameters, suggesting that BCAA may reverse positive exercise effects. Of note, BCAA supplementation even promoted neuroinflammation in brain regions that were unaffected by HFD feeding. In contrast, exercising mice treated with dietary BCAA exhibited brain-region-specific improvements in vascular density as well as improved myelin quality in the hippocampus. Furthermore, we found beneficial effects of exercise and BCAA supplementation on metabolism and behavior. BCAA and exercise decreased body weight, increased the percentage of eWAT and hind leg muscle in the body, decreased fasting insulin levels, and improved the circadian rhythm. Interestingly, only exercising mice with dietary BCAA exhibited improvements in spatial learning and memory. Furthermore, exercise and BCAA transiently increased muscle strength after 1 month of treatment.

### 4.1. Metabolism and Physical Activity

After 6 months of HFD feeding, severe obesity manifested in all Ldlr−/−.Leiden mice, as they displayed increased body weight, white adipose tissue accumulation, hypercholesterolemia, hypertriglyceridemia, and insulin resistance, as previously in other studies using Ldlr−/−.Leiden animals [40,41,42,48,77,78,79]. Interestingly, in this study, exercising mice on BCAA gained less body weight than both exercising and non-exercising mice, while the body fat percentage was similar across groups. However, compared to exercising mice that did not receive BCAA supplementation, mice that both exercised and received BCAA supplementation exhibited a higher eWAT mass as a proportion of their total body weight. This result differs from the effects of isoleucine or valine monotreatment in Ldlr−/−.Leiden mice on a fructose-containing HFD [80]. In contrast, Zhang et al. demonstrated that BCAA supplementation attenuated HFD-induced weight gain and decreased adipose tissue accumulation at the expense of abnormal lipolysis [81]. In this study, reduced body mass was not related to differences in energy consumption, as caloric intake was similar between groups. It has previously been posited that higher protein intake, particularly leucine, is associated with lower body weight due to browning and heat production in thermogenic tissues, resulting in increased resting energy expenditure [82,83]. 

Activity-related energy expenditure may contribute to differences in body weight. Subtle differences in home-cage activity patterns were detected in DVC. Different research groups have investigated the effect of BCAA supplementation on endurance performance [84,85,86]. Although results are inconsistent, a recent meta-analysis shows that BCAA ingestion has favorable effects on energy metabolism, reduces fatigue substances and muscle soreness, and delays central fatigue [37,87]. A limitation of this study was the high variability in total running distance of pair-housed mice per home cage, as we employed a voluntary exercise paradigm. Forced exercise, in turn, causes higher levels of stress and is therefore less translational [88,89]. Importantly, we found that exercise in combination with BCAA supplementation influenced the animals’ circadian rhythm. BCAA-supplemented mice had higher nighttime home-cage activity, but lower daytime home-cage activity compared to mice without BCAA. Likewise, we measured lower individual locomotor activity and spontaneous exploratory behavior in the BCAA-fed mice during a daytime open field test. Previous research has demonstrated that HFD can dampen the diurnal rhythm of locomotor activity by altering molecular and metabolic processes that change the circadian clock [90]. Our results show that exercising and BCAA-supplemented mice were more active at night, possibly by attenuating HFD-related alterations of the circadian rhythm. Ultimately, increased home-cage activity may have contributed to higher activity energy expenditure, which may be causal for decreased body weight. 

Next to endurance performance, BCAA, and leucin in particular, play a key role in muscle protein synthesis. Of note, recent research has shown that BCAA supplementation has no additive effect on muscle strength and hypertrophy in healthy individuals [91]. We found that the total gastrocnemius and soleus muscle mass increased only in exercising mice compared to non-exercising mice; however, the percentage of gastrocnemius and soleus mass relative to body weight was higher in the exercising mice that received BCAA supplementation. This did not translate into improved fine motor coordination and balance, as measured by the rotarod test. In contrast, the rotarod performance decreased with increasing severity of obesity, as observed in previous studies [58]. However, our data showed that exercise combined with BCAA supplementation significantly increased total grip strength after one month of treatment, which may be facilitated by beneficial effects on ATP generation in the muscle [92]. In the present study, it needs to be considered that a chronic HFD-diet intake leads to nonalcoholic fatty liver disease (NASH) and hepatic steatosis in Ldlr−/−.Leiden mice [40,41,80]. Hepatic dysfunction is associated with muscle depletion and decreased strength, which can be counteracted by dietary BCAA supplementation [93,94,95,96]. In addition, we found that combined treatment with BCAA and exercise, but not exercise alone, prevented severe HFD-induced hyperinsulinemia, which is a measure of insulin resistance. This profound metabolic effect can thus be attributed to BCAA supplementation, consistent with previous research that found improvements of manifested obesity and hyperinsulinemia in Ldlr−/−.Leiden animals fed a fast-food diet and given comparable doses of single BCAA [80]. Our data showed that exercise in combination with BCAA supplementation prevented body weight gain, most likely facilitated by increased energy expenditure due to improved circadian rhythm. 

### 4.2. Cerebrovasculature

Obesity can cause vascular and metabolic dysfunctions that increase the risk of atherosclerosis and hypertension. All experimental groups exhibited hypertriglyceridemia and hypercholesterolemia, which promote atherosclerosis in Ldlr−/−.Leiden mice—a major risk factor for hypertension [39]. In line with previous studies, HFD feeding increased systolic blood pressure in the Ldlr−/−.Leiden mice, which is a major risk factor for hypertension and reduced CBF [40,41,97]. After 3.5 months of HFD feeding, CBF levels started to decrease in the cortex, hippocampus, and thalamus. After 6 months of treatment, cortical and thalamic CBF dropped below baseline CBF levels, likely due to hypertension [97]. Therefore, our findings are consistent with previous animal and human studies that show that obesity is linked to cerebral hypoperfusion [6,55,97]. The effects of exercise on CBF in animal models of obesity have hardly been studied; however, physical activity increases CBF in healthy mice [98]. Unexpectedly, hippocampal CBF in both exercise groups decreased below baseline levels and was significantly lower than in non-exercising animals after 6 months of treatment. We hypothesize that the CBF of the HFD-treated Ldlr−/−.Leiden mice stabilized with increasing obesity burden, reflecting a potential compensatory hemodynamic mechanism that protects against early pathological changes. In previous studies, using non-exercising HFD-induced obese Ldlr−/−.Leiden mice, regional CBF even increased [48]. Similar compensatory mechanisms have been discovered in the preclinical phase of Alzheimer’s disease [99,100].

In addition, HFD + exercise mice also reacted differently under vasoconstrictive conditions. Cortical and hippocampal CBF decreased during 6 months of treatment, whereas no changes were measured in the HFD and HFD + BCAA + exercise groups. It is possible that the vasculature of exercising mice without BCAA responded better to vasoconstrictive stimuli, indicating a healthier vasculature despite the pronounced atherosclerosis development in the HFD-fed Ldlr−/−.Leiden mice [39]. However, these adaptations were subtle, as the vasoreactivity remained unchanged from baseline in all experimental groups. Further work is required to establish how exercise remodels the cerebrovascular network, e.g., exercise may promote angiogenesis or attenuate atherosclerosis. 

Obesity and HFD feeding cause impairments in cerebral microvasculature, leading to cerebral hypoperfusion [101]. Vascular density in the total brain, measured by a relative glucose transporter 1 (GLUT-1) positive area, was unaffected after 6 months of HFD feeding. Of note, both exercise groups were able to counteract the HFD-induced decrease of vascular quality (amount of GLUT-1) in the cortex and hippocampus. A previous study in mice has shown that increased energy demand during exercise promotes the expression of GLUT-1 transporters in the cortex, although this acute effect diminished after 24 h [102]. Interestingly, the BCAA intervention strongly increased cortical vascular density compared to the other treatment groups and even exceeded the vascular density measured in the young chow-fed control mice. Moreover, the BCAA-supplemented mice had increased vascular density in the external capsule as well as in the basal ganglia—a region responsible for the control of voluntary motor movements. The influence of BCAA on GLUT-1 transporters in the cerebral capillaries has not been investigated. Recently, Iwai et al. reported that BCAA enhanced ATP production by promoting glucose uptake through translocation of GLUT-1 transporters to the plasma membrane of cultured cells [92]. Furthermore, BCAA supplementation was found to increase vascular endothelial growth factor (VEGF) levels in the blood after exercise in humans. VEGF is a signaling protein that promotes angiogenesis [103]. In this study, increased vascular density in BCAA-supplemented mice did not translate into improvements in CBF, suggesting that vascular remodeling had not yet established a functional capillary network [104]. Nevertheless, this is the first study to demonstrate region-specific improvements in vascular integrity after oral BCAA supplementation. Supposedly, BCAA-induced changes in glucose metabolism may contribute to increased vascular density in the brain—a mechanism that should be more closely studied in future.

Hypertension and subsequent changes in microvasculature as well as decreased CBF can cause disruptions in functional connectivity (FC) between brain changes by directly affecting blood-oxygen-level-dependent (BOLD) signaling [105]. In coherence with HFD-induced hypoperfusion and declined vascular quality, we also found compromised FC (total correlations) between multiple brain regions not being altered in the exercise groups. Together, we demonstrated subtle exercise-related improvements in vascular health, as the ability to respond to vasoconstrictive stimuli was enhanced. For the first time, we demonstrated that exercise in combination with BCAA supplementation increased region-specific vascular density. Exercise (+BCAA) intervention did not attenuate the obesity-related decrease of functional connectivity between multiple brain regions.

### 4.3. White and Grey Matter Integrity

There is growing evidence that obesity is associated with alterations in WM, with different research groups reporting both increased WM volume and WM atrophy [106]. In this study, in vivo diffusion tensor imaging revealed that obese mice, regardless of the treatment group, displayed increased fiber integrity, as measured by fractional anisotropy (FA), in the GM and WM, and subregions, including the corpus callosum, basal ganglia, fornix, corpus callosum, and optic tract after 3.5 months of HFD feeding. Mean diffusivity (MD), in turn, was decreased in the white matter and subregionally in the somatosensory cortex, basal ganglia, and the optic tract. These findings may be related to age-related increase in white matter volume [107]. Until middle age (40 years of age), white matter is increases steadily in lean and obese people [107]. The ages of the mice in the present study ranged from adolescent mice (4 months of age) at baseline, to young adulthood (7.5 months of age) at 3.5 months after treatment, to middle age (10 months of age) at 6 months after treatment [107,108]. Hence, the initial increase in fiber integrity in young adulthood is unaffected by obesity, but middle age appears to be a period of vulnerability to the effects of obesity on brain structure [107]. Accordingly, we found that exercise prevented WM loss in the corpus callosum and fornix between 3.5 months and 6 months of treatment. A recent meta-analysis found that obesity is particularly associated with reduced WM integrity in the corpus callosum—the largest interhemispheric WM connection that is involved in multiple motor, sensory, and cognitive functions of the brain [7]. Post mortem quantification of myelin density and quality using polarized light imaging (PLI) showed no evidence of myelin or axonal damage in the HFD-fed mice compared with the exercising mice, most likely due to the early stage of obesity. The age-related increase in myelin density was still present in different subregions in both the exercising and non-exercising mice post mortem after 6 months of treatment. Of note, increased myelin density was not measured in the BCAA-supplemented animals. To summarize, our data reflected an age-related increase in white and grey matter volume from the age of 3 to 7.5 months. At middle-age (10 months), exercise prevented the HFD-induced decreases in WM integrity.

### 4.4. Neuroinflammation

The exact mechanism by which obesity leads to WM changes, and how exercise may be able to prevent WM damage, remains unknown. Animal studies suggest that a chronic state of systemic inflammation, associated with adipose tissue pathogenesis and dysfunction, plays a critical role in alterations of GM and WM in the brain [109,110]. HFD may induce disruption of the blood–brain barrier and elevated levels of inflammatory markers, which in turn may trigger structural changes in the brain [111].

Consistent with other studies using diet-induced obese Ldlr−/−.Leiden mice, we found increased neuroinflammation in several brain regions (basal ganglia, cortex, hippocampus, and thalamus) of the HFD-fed mice [40,42]. Importantly, 6 months of exercise prevented neuroinflammation in the cortex, corpus callosum, and fimbria. In agreement, other animal studies have shown that exercise reduces obesity-related proinflammatory markers and normalizes glial cell activation [112]. Likewise, physical activity also reduced inflammation markers in obese children [113]. Together, obesity-related decrease in regional WM integrity may be ameliorated by the anti-inflammatory effects of exercise. On the contrary, exercising mice treated with BCAA did not recapitulate the beneficial exercise effects. BCAA supplementation even induced increased IBA-1 expression in regions that were unaffected by HFD feeding, including the fimbria and external capsule. Additionally, BCAA-supplemented mice displayed elevated neuroinflammation in the corpus callosum, cortex, and internal capsule compared to exercising animals without BCAA, suggesting that BCAA supplementation counteracts the positive effects of voluntary exercise. To our knowledge, this is the first study to investigate the effect of BCAA in the context of obesity and exercise on the brain. A study by De Simone et al. demonstrated that microglia cells in a BCAA-rich medium established a low level of chronic inflammation and exhibited decreased neuroprotective functions [33]. A previous study has indicated that BCAA can initiate inflammation and oxidative stress in endothelial cells and the vasculature, which may lead to endothelial dysfunction and potentially contribute to cardiovascular disease [114]. Future studies should include additional markers of (vascular) inflammation (e.g., GFAP) to provide a more complete understanding of the underlying mechanisms of neuroinflammation and how it is affected by exercise and BCAA [12]. For example, GFAP expression has been shown to increase in astrocytes in response to inflammation [12]. In this study, the BCAA-supplemented exercise-treated mice behaved similarly to the HFD-fed mice with respect to the majority of neuroimaging parameters assessed, possibly because BCAA reversed exercise-induced beneficial effects on the brain.

A limitation of the current study is that we did not measure BCAA levels in the animals, but we expected that BCAA levels would not be extensively increased, as exercise is known to reduce circulating BCAA [115,116]. Together, the exercise attenuated HFD-induced neuroinflammation. Additionally, it has been shown for the first time that BCAA supplementation reverses the beneficial effects of exercise in the brain and induces IBA-1 expression in brain regions unaffected by HFD feeding.

### 4.5. Cognition

Obesity is acknowledged to be a risk factor for cognitive impairment, particularly affecting hippocampal structure and function [2]. In this study, spatial learning was not affected after 5 months of HFD feeding, as all mice were able to find the platform position in the MWM. Only exercising mice that were treated with BCAA performed slightly better than the non-exercising animals. Likewise, in the probe trial, spatial memory was improved in the exercising mice on BCAA compared to the non-exercising mice, but not compared to the exercising mice without BCAA. Several studies have confirmed that exercise improves cognition, facilitated by mechanisms such as improved neurogenesis and neuroplasticity [117]. In contrast, we did not find any effects of exercise (+BCAA) on neurogenesis, as the amount of doublecortin-positive cells in the hippocampus was similar between groups. In the future, analysis of mature neurons (e.g., NeuN) may provide additional insight into potential treatment effects of exercise and BCAA against obesity-induced neuronal loss [118]. The effects of BCAA supplementation on cognition are largely unknown. A preclinical study showed that BCAA promoted cognitive improvement by restoring hippocampal function after a traumatic brain injury, although the mice used were in a BCAA-deficient state [119]. Regarding other neuroimaging parameters, BCAA supplementation did not ameliorate HFD-induced changes in functional connectivity, CBF, or WM and GM integrity. On the contrary, we measured reduced hippocampal fiber dispersion in these mice, indicating improved myelin quality. Moreover, we found improved vascular integrity in cortical regions that played a critical role in spatial memory tasks in exercising and BCAA-supplemented animals [120]. In addition, Park et al. reported that exercise improved cognitive function by ameliorating obesity-induced insulin resistance in HFD-fed C57BL/6 mice. In the brain, insulin is directly involved in memory consolidation [121]. Taken together, we found that BCAA reduced hyperinsulinemia and, supposedly, a combination of both exercise and dietary BCAA may have a synergistic effect in attenuating obesity-related cognitive impairment.

### 4.6. Limitations

The main limitation of the study was the lack of a control group receiving only BCAA supplementation, as the current design only allowed for the evaluation of BCAA supplementation in combination with voluntary exercise. Furthermore, we sacrificed young non-obese mice after taking baseline measurements and used the harvested tissues as a healthy non-obese reference group to compare with the tissues of the different treatment groups after a 6-month study period. However, this approach did not allow us to distinguish between treatment effects and aging effects. Similarly, for in vivo parameters, we used the baseline measurements of young non-obese mice as a reference, but we did not have an age-matched non-obese control group for the outcomes after the start of the intervention. Moreover, the use of the running wheel decreased during the last 2 months of the experiment, which may explain the transient nature of certain exercise (+BCAA) benefits (e.g., grip strength). Higher exercise intensities may have resulted in more pronounced effects on the brain. Obese mice appear to mimic human behavior in the sense that behavioral changes are difficult to maintain over long periods of time.

## 5. Conclusions

Our data suggest that voluntary exercise ameliorated neuroinflammation and regional loss of WM integrity, and improved CBF under vasoconstrictive conditions. In contrast, exercise could not prevent the onset of obesity-related metabolic risk factors, consistent with the view that exercise alone is not sufficient to prevent all detrimental effects of obesity. For the first time, we have shown that BCAA and exercise do not synergistically prevent HFD-induced neuroinflammation and abnormalities of brain structure and function, which may indicate that BCAA may reverse the beneficial effects of exercise. In contrast, our data imply that BCAA may have beneficial effects on HFD-induced dysmetabolism. Furthermore, exercising mice treated with BCAA exhibited slightly improved cognition and transiently improved grip strength. In future, it is necessary to investigate whether dietary supplementation with BCAA alone can yield comparable beneficial effects. Based on the results, BCAA supplementation should be used with caution, as it may have harmful effects on the brain.

## Data Availability

The datasets generated for this study are available on request to the corresponding author.

## Supplementary Materials

The following supporting information can be downloaded at: https://www.mdpi.com/article/10.3390/nu15071716/s1, Figure S1: Rotarod; Figure S2: Cortical thickness and hippocampal volume; Figure S3: Cerebrovascular reactivity; Figure S4: (A) A high-resolution image of the negative control for GLUT-1 immunohistochemical staining and (B) a representative image of the brain section stained for GLUT-1 (2.5× magnification). Scale bar: 1 mm; Figure S5: Neurogenesis; Figure S6: White adipose tissue, body fat and muscle mass. Figure S7: Metabolic markers. Figure S8: Open field test. Figure S9: Grip strength test. Figure S10: (Reverse) Morris water maze. Figure S11: Novel object recognition test. Figure S12: Resting-state functional connectivity based on partial correlations. Figure S13: Neuroinflammation. Table S1: Ingredients and composition of special diets; Table S2: Imaging parameters. Table S3: Average area ± SEM of the regions of interest that were manually selected using the freehand tool in ImageJ group on the brain sections used for immunohistochemistry (IHC) and polarized light imaging (PLI). Table S4: Sample size (number of mice or cages) per experiment. Table S5: Excluded mice per experiment and reason for exclusion. Table S6: Summary statistics of body weight, caloric intake, muscle weight, and fat tissue analysis. Table S7: Summary statistics of fasting blood and plasma markers and systolic blood pressure analysis. Table S8: Summary statistics of DVC metrics and open field parameters. Table S9: Summary statistics of the grip strength test and Rotarod. Table S10: Summary statistics of the (reverse) Morris Water Maze. Table S11: Summary statistics of the resting-state functional connectivity analysis based on total correlations. Table S12: Summary statistics of the resting-state functional connectivity analysis based on partial correlations. Table S13: Summary statistics of cerebral blood flow analysis. Table S14: Summary statistics of cerebral blood flow analysis. Table S15: Summary statistics of Diffusion tensor imaging analysis. Table S16: Summary statistics of Polarized light imaging analysis. Table S17: Summary statistics of hippocampal volume and cortical thickness analysis. Table S18: Summary statistics of immunohistochemical analysis of ionized calcium-binding adapter molecule (IBA-1). Abbreviations: high-fat diet (HFD), branched-chain amino acids (BCAA).
