# Supplementary material for: The Preventive Effect of Exercise and Oral Branched-Chain Amino Acid Supplementation on Obesity-Induced Brain Changes in Ldlr−/−.Leiden Mice
Source: Nutrients. 2023 Mar 31;15(7):1716. doi: 10.3390/nu15071716 (PMC10097391; doi:10.3390/nu15071716)
Supplement: Supplementary file 1 [file nutrients-15-01716-s001.zip › nutrients-2256139-supplementary.pdf]

Supplementary Materials and Methods

Novel object recognition test

The ORT was performed in 3 consecutive days, consisting of the habituation phase, the familiarization phase, and the testing phase. First, animals were habituated to an empty open field box with transparent walls (45 x 45 x 30 cm) for 4 min. For 3 consecutive days the ORT was repeated with increasing inter-trial intervals between the familiarization and testing phases (30 min on day 1, 1h on day 2, and 2h on day 3), performed as follows: during the familiarization phase, two identical objects (calcium eggs, tea-light holders, yellow plastic ice cream cones, or bottles filled with sand) (F1 and F2) were placed equidistant from the center of the box. After 4 min of free exploration, the mice returned to their home-cage. Thirty minutes later, mice were placed into the same arena, with one of the familiar objects (F3) and one novel object (N1) that differed in shape and appearance (testing phase). Again, each mouse was allowed to explore the objects for 4 minutes. The position of the novel object (i.e., left or right) was counterbalanced across trials.

Exploratory behavior of the mice was measured using EthoVision XT 16 (Noldus, Wageningen, the Netherlands) as direct contact with the object, or movement within a 2cm diameter around the object. Several indexes were calculated that describe the relationship between the exploration of the familiar objects and subsequent object recognition of the novel object during the testing phase. First, the recognition index reflects the percentage of time spent exploring the novel object (N1) relative to the total time spent exploring both, the novel (N1) and the familiar object (F3) (RI = N1/(N1 + F3)). Second, the discrimination index (DI) reflects the discrimination between the novel object (F3) and the familiar object (N1) (DI = (N1 − F3)/(N1 + F3)). The index between +1 and −1 is obtained. A positive result indicates that the animals spent more time around the novel object, while a negative score indicates that the mice spent more time exploring the familiar object, and a score of 0 indicates a null preference. Third, the preference index for any one of the two objects (novel (N1) or familiar (F3)) was calculated as proportion of the total time spent exploring both objects (PI = 100 × ([N1 or F3]/(N1 + F3)). When N1 is the numerator, a value closer to 100% indicates a preference for N1, 50% indicates no preference, and less than 50% indicates a preference for F3 (vice versa when F3 is the numerator).

Polarized light imaging

Different PLI maps were derived from the raw images by fitting them to the Jones formula: 1) transmittance, (2) retardance, (3) in-plane, (4) inclination, and (5) FOM-HSV map [1, 2]. The transmittance map resembles the average light intensity across all polarizer orientations and thereby represents the absorption of light by the brain tissue [1, 2]. On the obtained images, the myelin dense white matter appears dark, while grey matter appears brighter. The

retardance map measures the magnitude of the phase shift induces to the light wave because of birefringent tissue. Assuming that most of the birefringence in nervous tissue arises from myelin, PLI is an indirect measure for myelin density[3]. Therefore, a decrease in retardance is indicative for myelin loss. Importantly, the retardance map can also be influenced by tissue thickness, inclination angle (i.e., the out-of-plane angle in the third dimension) of the myelinated fiber, and the local birefringent properties of myelin. Furthermore, crossing fiber (orthogonal) cancel out the birefringence effect, which results in lower retardance values. The in-plane orientation map describes the in-section direction angle of each myelinated fiber (x-y orientation) [2]. The inclination map measures the out-of-section angle because it measures the vertical component of each fiber [2]. Finally, the fiber orientation map (FOM)-HSV map is a color-coded adaptation of the retardance map and the in-plane orientation map. The FOM is color-coded representation of the direction of fibers with its intensity modulated by the myelin density [3].

Supplementary Results

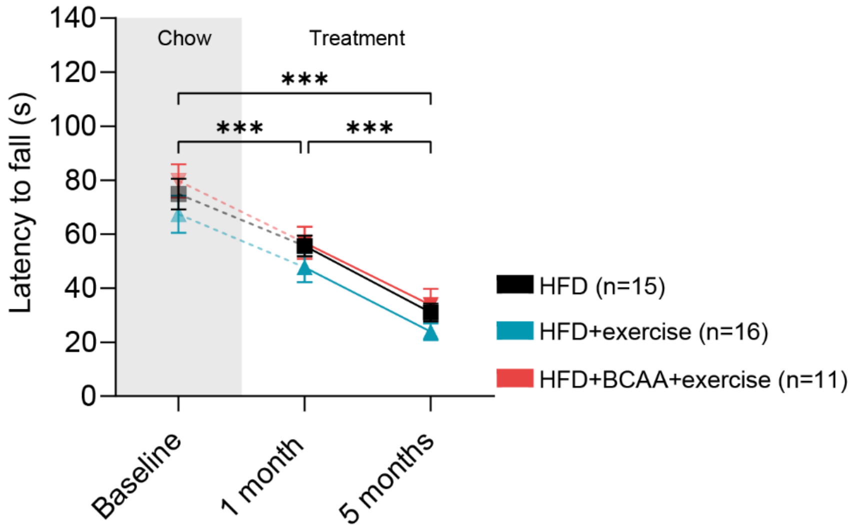

Supplementary Figure S1. Rotarod. The rotarod test was conducted during baseline measurements and again after 1 month and 5 months of treatment to assess the impact of HFD, HFD+exercise, and HFD+BCAA+exercise on motor balance. In all experimental groups, the latency to fall decreased, and after 6 months of treatment, the latency to fall was even lower than during baseline measurements. However, no significant treatment effects were observed. High-fat diet (HFD), branched-chain amino acids (BCAA). Data are presented as mean ± SEM. *** $p < 0.001$.

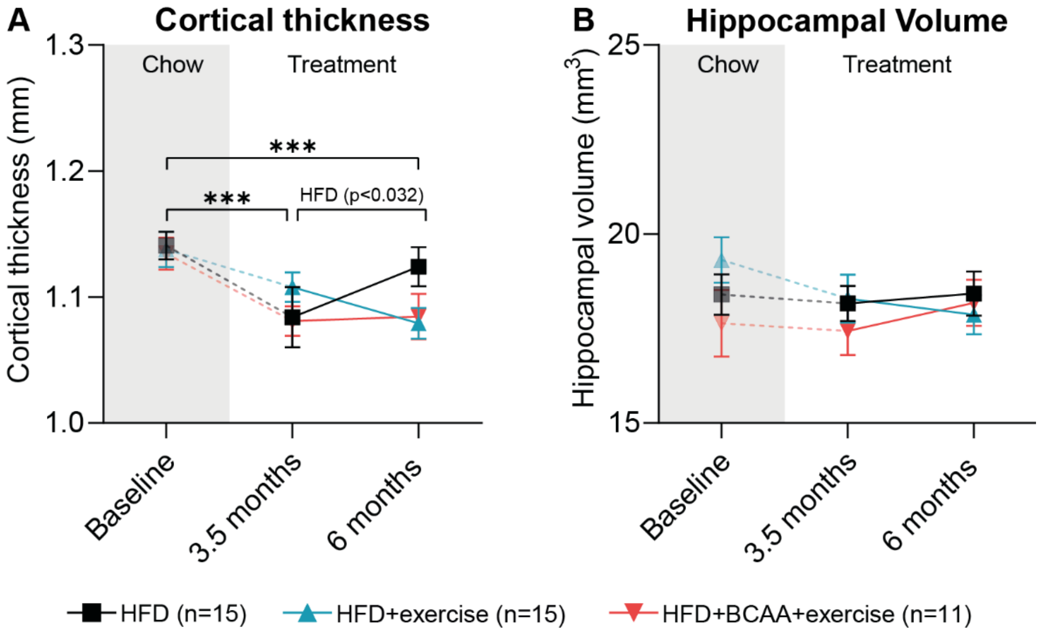

Supplementary Figure S2. Cortical thickness and hippocampal volume. (A) Cortical thickness and (B) hippocampal volume at baseline (chow) and after 3.5 and 6 months of treatment with HFD, HFD+exercise and HFD+BCAA+exercise. A decrease in cortical thickness was observed in all mice between baseline and 3.5 months of treatment and between baseline and 6 months of treatment. Only HFD-fed mice showed an increase in cortical thickness between 3.5 and 6 months of treatment. No changes in hippocampal volumetry were detected. High-fat diet (HFD), branched-chain amino acids (BCAA). Data are presented as mean ± SEM. ***p < 0.001.

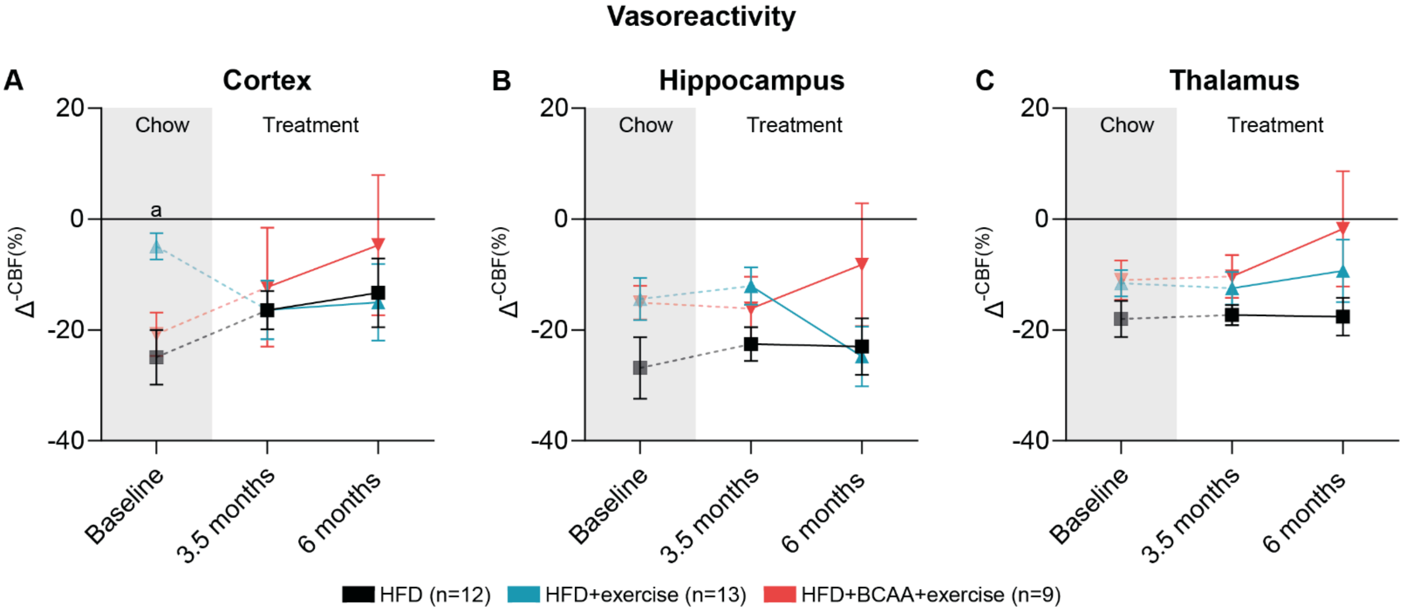

Supplementary Figure S3. Cerebrovascular reactivity. Cerebral blood flow (CBF) was measured by arterial spin labeling during baseline (chow) and after 3.5 and 6 months of treatment with HFD, HFD+exercise and HFD+BCAA+exercise. Vasodilative and vasoconstrictive CBF levels were used to calculate cerebral vasoreactivity in the cortex (A), hippocampus (B) and thalamus (C). High-fat diet (HFD), branched-chain amino acids (BCAA), cerebral blood flow (CBF). Data are presented as mean ± SEM. a: significant difference HFD vs. HFD+exercise group.

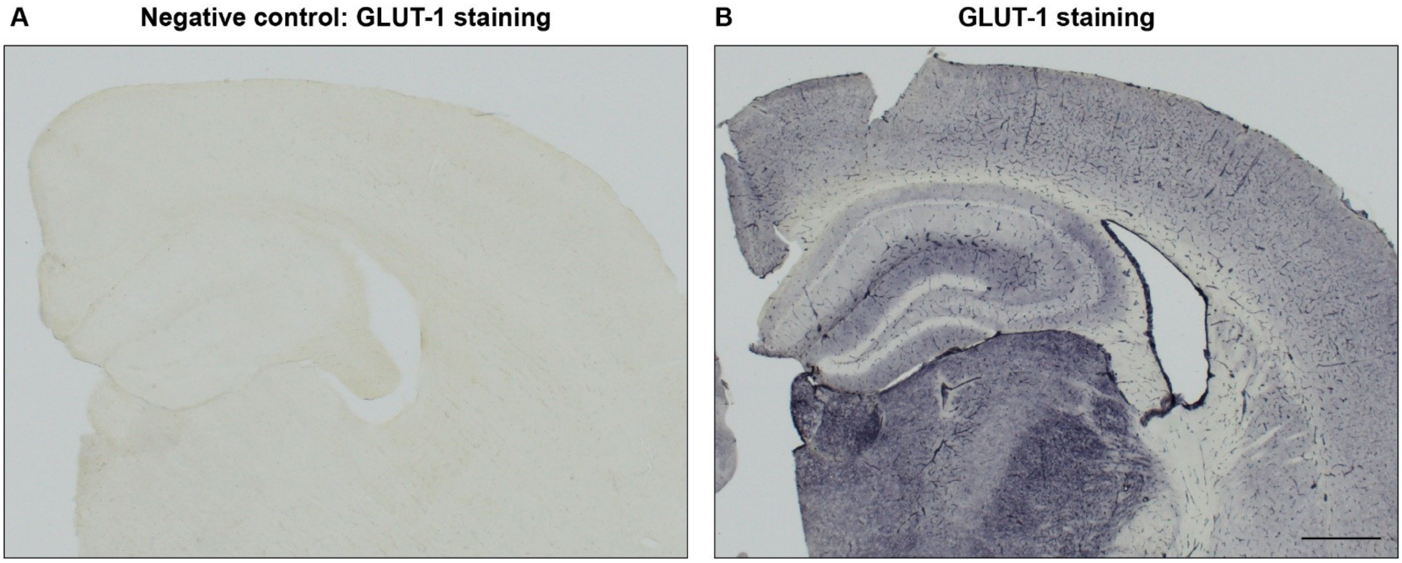

Supplementary Figure S4. (A) A high-resolution image of the negative control for GLUT-1 immunohistochemical staining and (B) a representative image of the brain section stained for GLUT-1 (2.5× magnification). Scale bar: 1 mm.

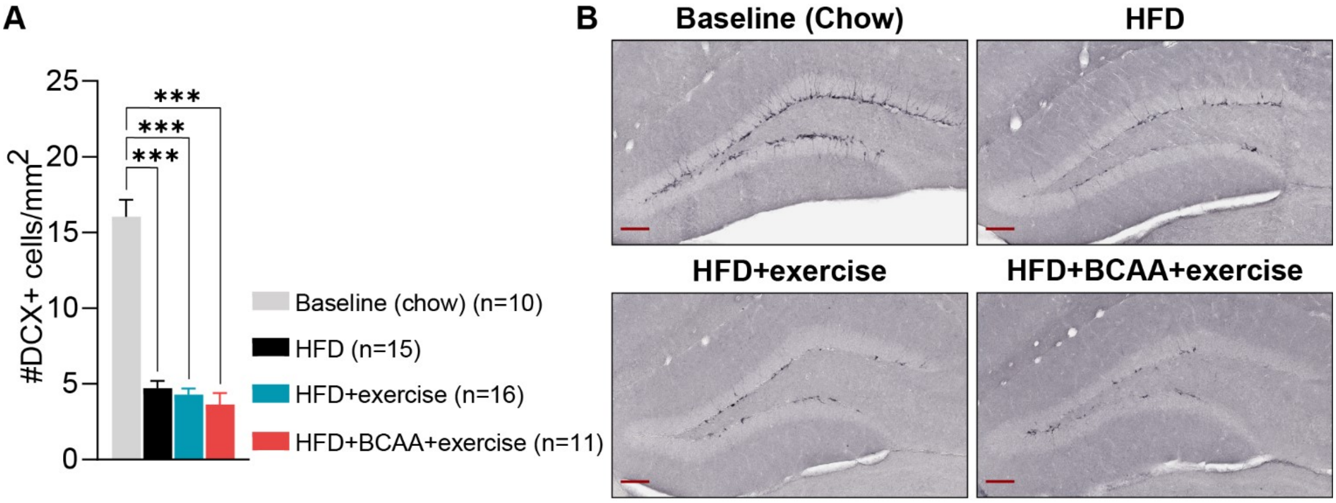

Supplementary Figure S5. Neurogenesis. (A) Immunohistochemical staining for newly formed neurons by staining doublecortin (DCX) in the dentate gyrus of the hippocampus of young chow-fed animals (baseline), HFD, HFD+exercise, and HFD+BCAA+exercise mice. Neurogenesis was higher in young chow-fed mice at baseline measurements compared to the other treatment groups after 6 months of treatment. No other treatment differences were observed. (B) Representative images of the DCX staining in the dentate gyrus (scale bar = 100 µm). High-fat diet (HFD), branched-chain amino acids (BCAA). Data are presented as mean ± SEM. *** $p < 0.001$.

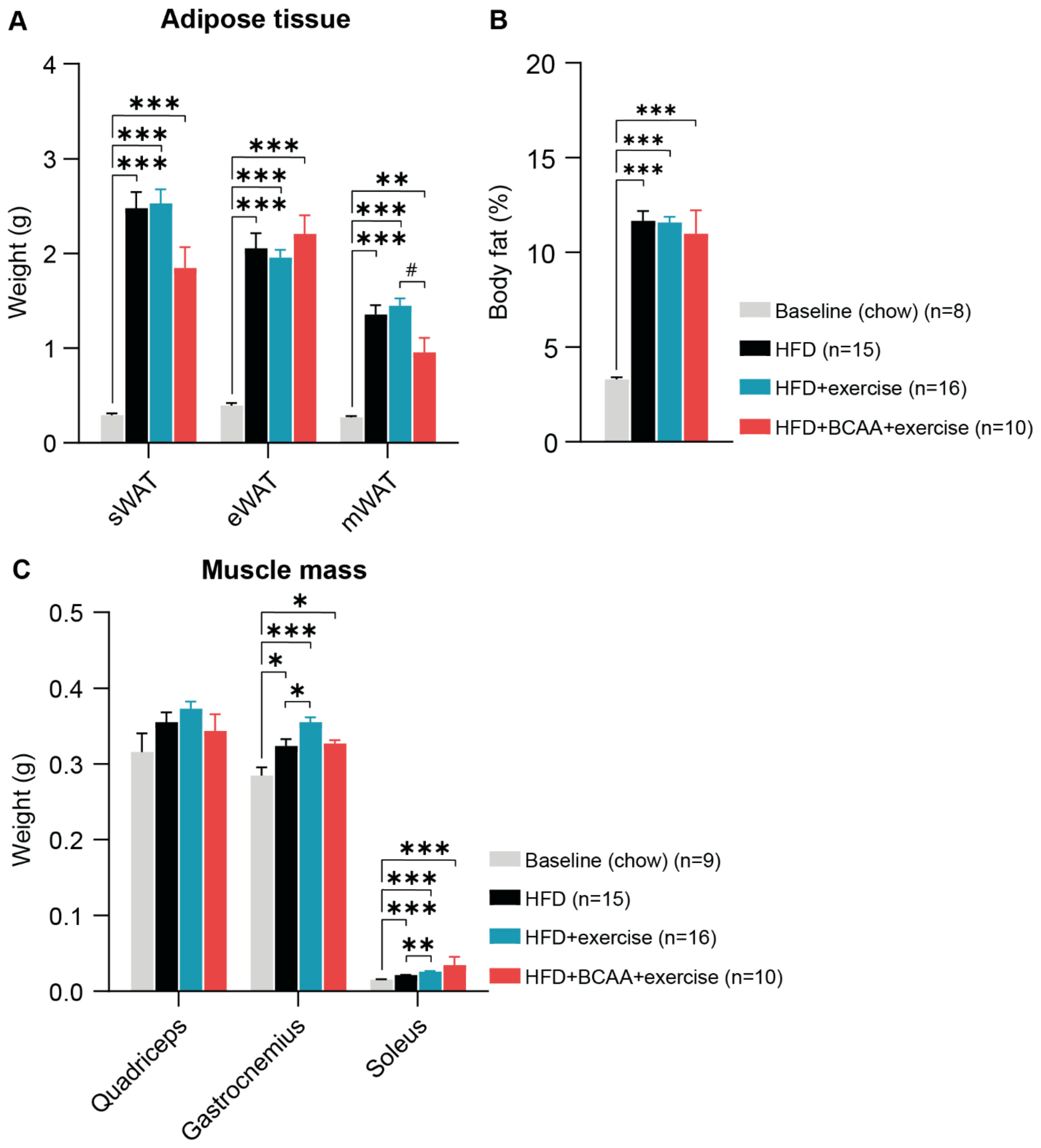

Supplementary Figure S6. White adipose tissue, body fat and muscle mass. (A) Weights of white adipose tissue depots (subcutaneous adipose tissue (sWAT), epididymal white adipose tissue (eWAT), mesenteric white adipose tissue (mWAT)) and (B) weights of the hind leg muscles (quadriceps, gastrocnemius, soleus) measured in young chow-fed mice and mice treated with HFD, HFD+exercise, or HFD+BCAA+exercise for 6 months. All HFD-fed mice developed adiposity in the different fat depots. In HFD+BCAA+exercise mice mWAT mass tended to be less heavy than in the HFD+exercise group. In all mice, the gastrocnemius and soleus muscle mass was heavier after 6 months of treatment in comparison to young chow animals (Figure 2D). Notably, after 6 months of HFD feeding, exercising mice had a heavier gastrocnemius and soleus than non-exercising animals, whereas the combination of exercise with BCAA had no effect on muscle mass. (C) Body fat percentage increased in all HFD-fed mice compared to young chow-fed mice. High-fat diet (HFD), branched-chain amino acids (BCAA). Data are present as mean ± SEM. * $p < 0.05$, ** $p < 0.01$, *** $p < 0.001$, # $0.05 < p < 0.08$ (trend); b: significant difference HFD vs. HFD+BCAA+exercise group; c: significant difference HFD+exercise vs. HFD+BCAA+exercise group.

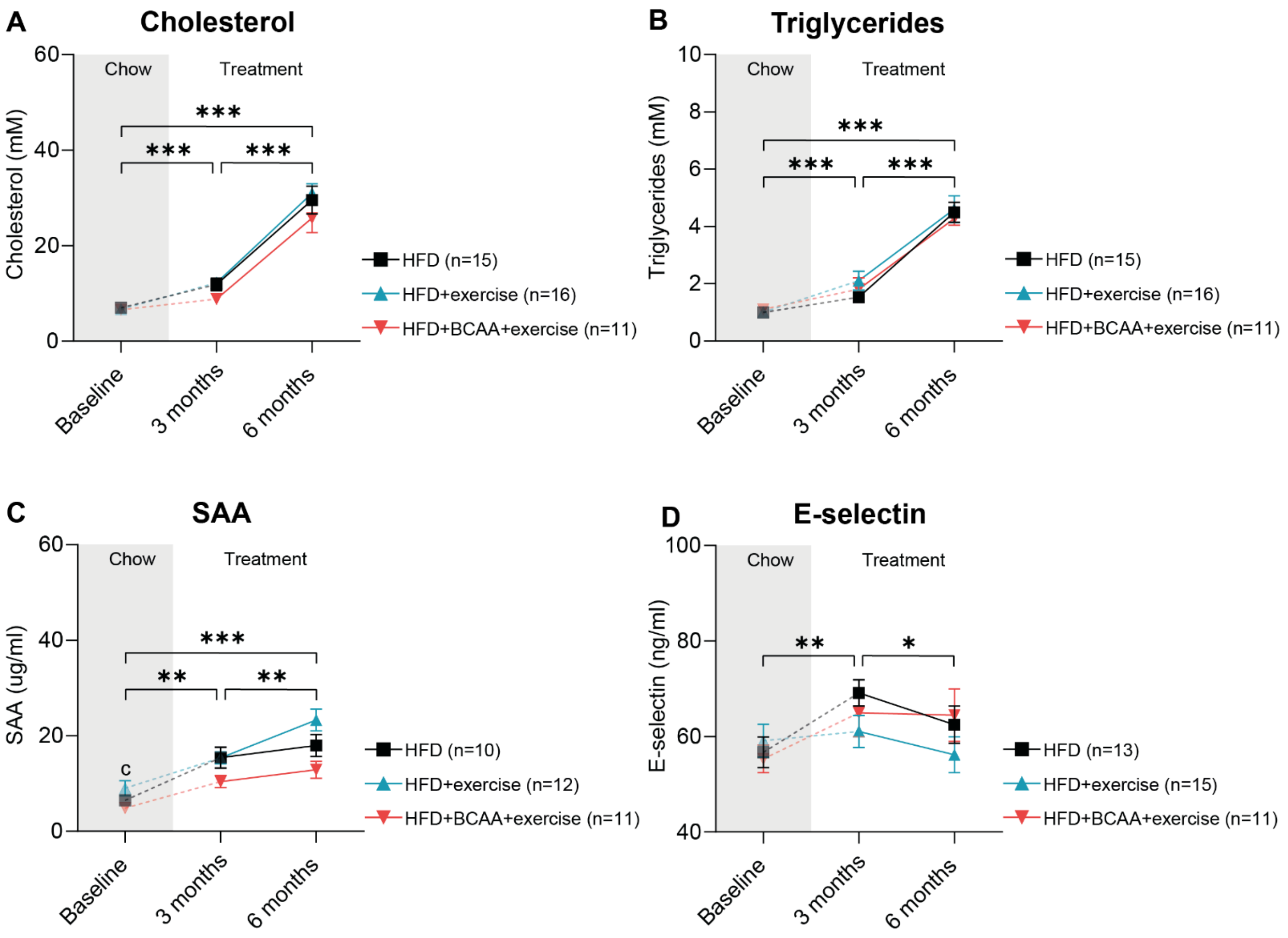

Supplementary Figure S7. Metabolic markers. (A) Cholesterol, (B) triglycerides, (C) serum amyloid (SAA), and (D) E-selectin were measured in the blood plasma of HFD, HFD+exercise, and HFD+BCAA+exercise treated animals at baseline (chow), and after 3 months and 6 months of treatment. Plasma levels of cholesterol, triglycerides, and serum amyloid A (SAA) increased steadily throughout the treatment period. After an initial increase in E-selectin after 2 months of treatment, plasma E-selectin levels decreased again, resulting in plasma levels comparable to the baseline measurements after 6 months of treatment. No significant treatment effect was measured for any of the markers. High-fat diet (HFD), branched-chain amino acids (BCAA). Data are presented as mean ± SEM. * $p < 0.05$, ** $p < 0.01$, *** $p < 0.001$.

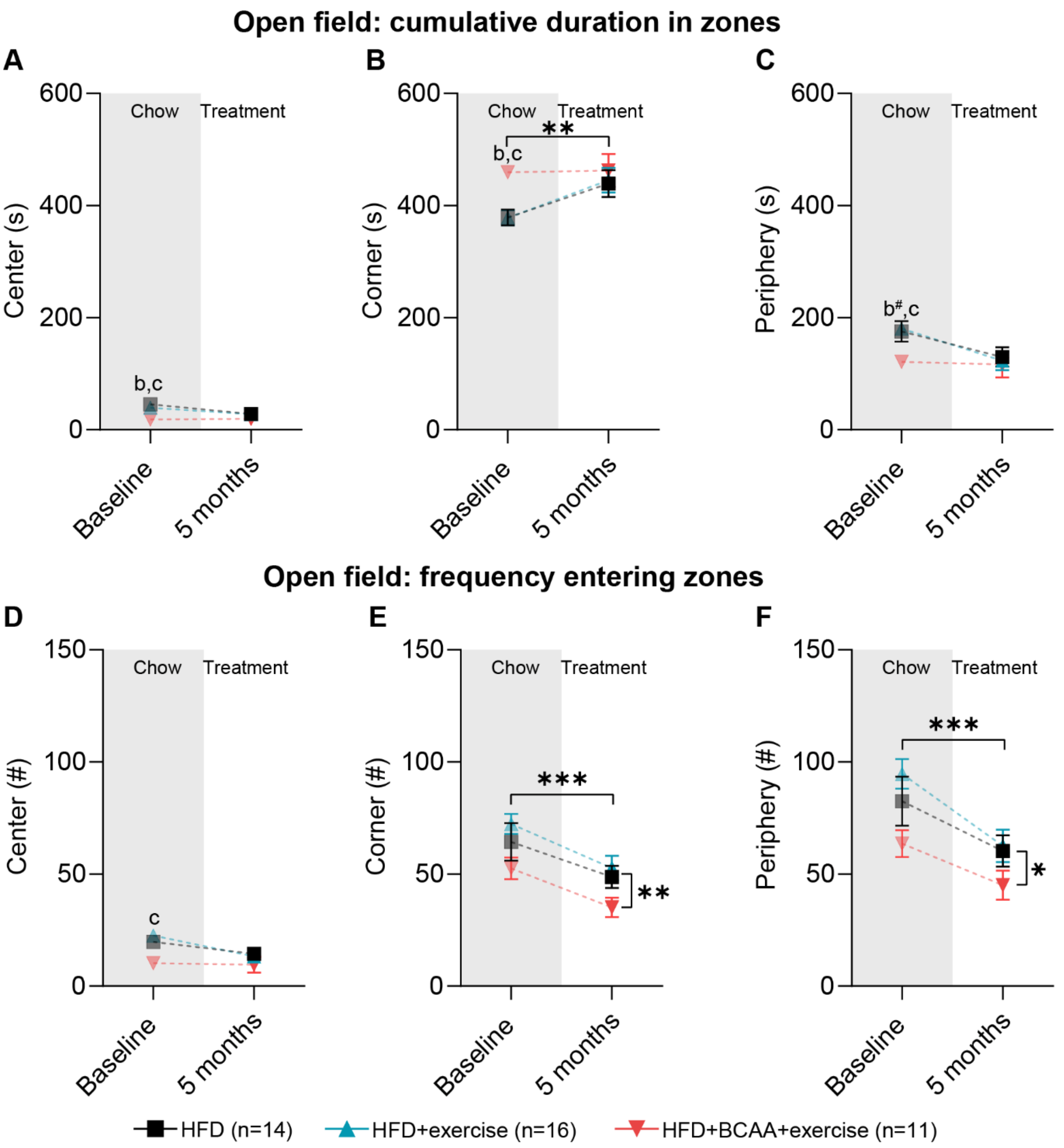

Supplementary Figure S8. Open field test. Spontaneous behavior and exploratory locomotion were measured at baseline measurements and again after 5 months of treatment with HFD, HFD+exercise, or HFD+BCAA+exercise. The cumulative time spent in the (A) center (B) corner (C) periphery and the frequency of entering the (D) center (E) corner (F) periphery of the open field arena were analyzed. After 5 months of treatment, all mice spent more time in the corners of the open field, and the frequency of mice entering the corners and periphery of the arena decreased. Notably, the frequency of entering the corners and periphery of the open field was significantly lower in the HFD+BCAA+exercise mice compared to the HFD+exercise mice. High-fat diet (HFD), branched-chain amino acids (BCAA). Data are presented as mean ± SEM. * $p < 0.05$, ** $p < 0.01$, *** $p < 0.001$, # $0.05 < p < 0.08$ (trend); b: significant difference HFD vs. HFD+BCAA+exercise group, c: significant difference HFD+exercise vs. HFD+BCAA+exercise group.

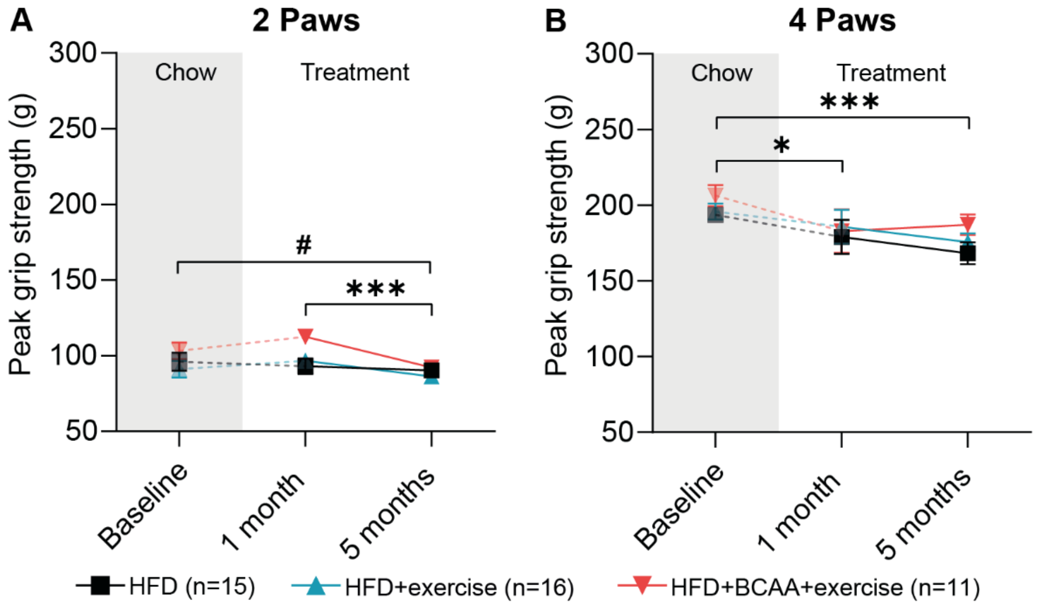

Supplementary Figure S9. Grip strength test. Total peak strength of (A) forelimbs (2 paws) and (B) fore- and hindlimbs together (4 paws) was measured at baseline (chow) and after 1 and 5 months of treatment with exercise (HFD+exercise) and exercise in combination with BCAA supplementation (HFD+BCAA+exercise). Forelimb peak force decreased between treatment months 1 and 5 in all groups. After 5 months of treatment, peak force was lower in all animals compared to baseline measurements. Peak grip strength of fore- and hindlimbs combined (4 paws) of all experimental groups started to decrease already after 1 month of treatment and was significantly lower after 5 months of treatment compared to baseline measurements. High-fat diet (HFD), branched-chain amino acids (BCAA). Data are presented as mean ± SEM. * $p < 0.01$, *** $p < 0.001$, # $0.05 < p < 0.08$ (trend).

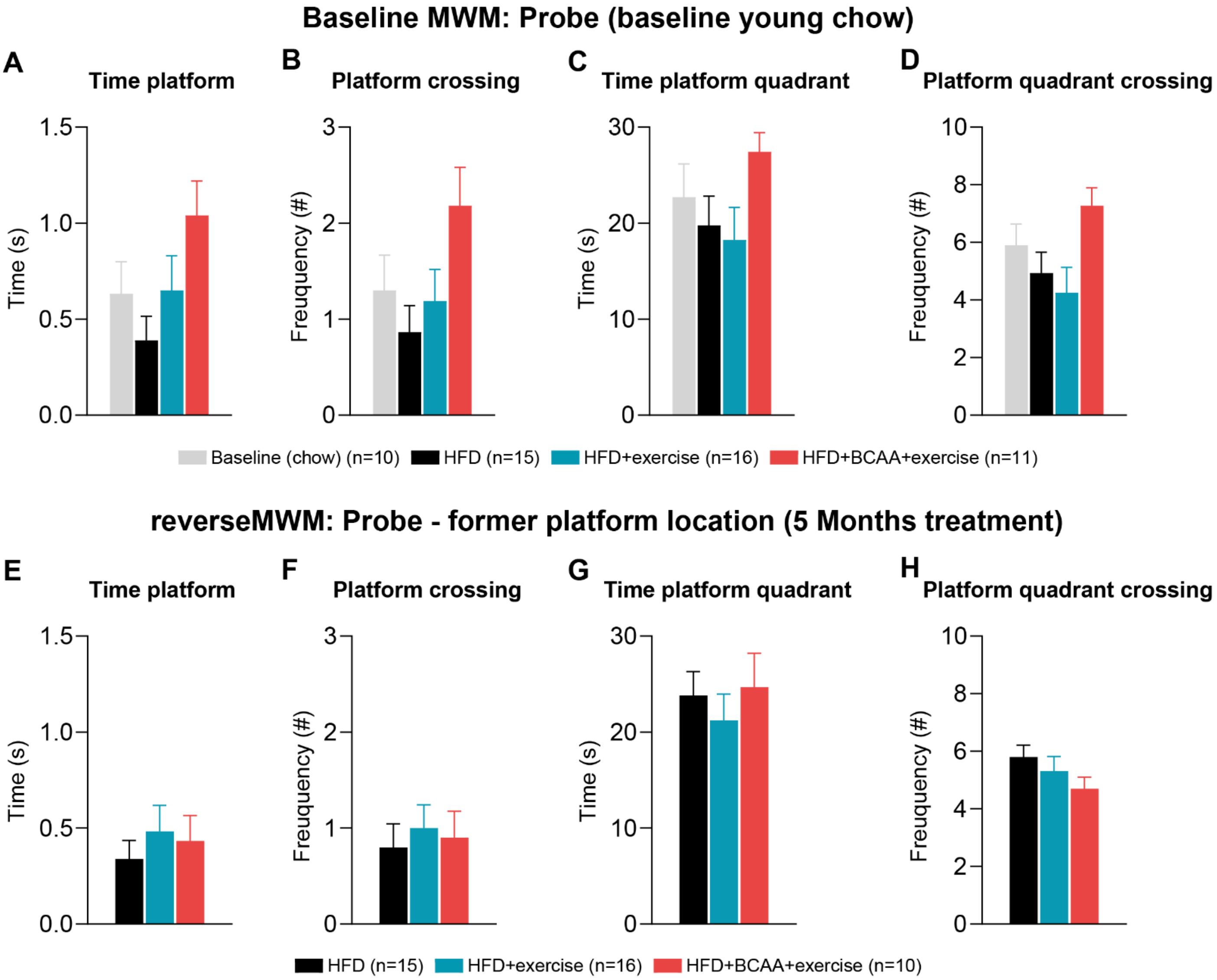

Supplementary Figure S10. (Reverse) Morris water maze. In a baseline Morris water maze (MWM) probe trial, the time animals spent in (A) the former platform location and (C) platform quadrant, as well as the frequency with which mice crossed (C) the former platform location and (D) the platform quadrant was assessed (platform location: north-east). After 5 months of treatment, the probe trial was repeated (reverse Morris water maze) and long-term spatial memory of the former platform location (north-east) was analyzed by again measuring the time the animals spent in (E) the former platform location and (G) the platform quadrant as well as the frequency the mice crossed (F) the former platform location and (H) the platform quadrant. No treatment differences were found. Data are presented as mean ± SEM. High-fat diet (HFD), branched-chain amino acids (BCAA).

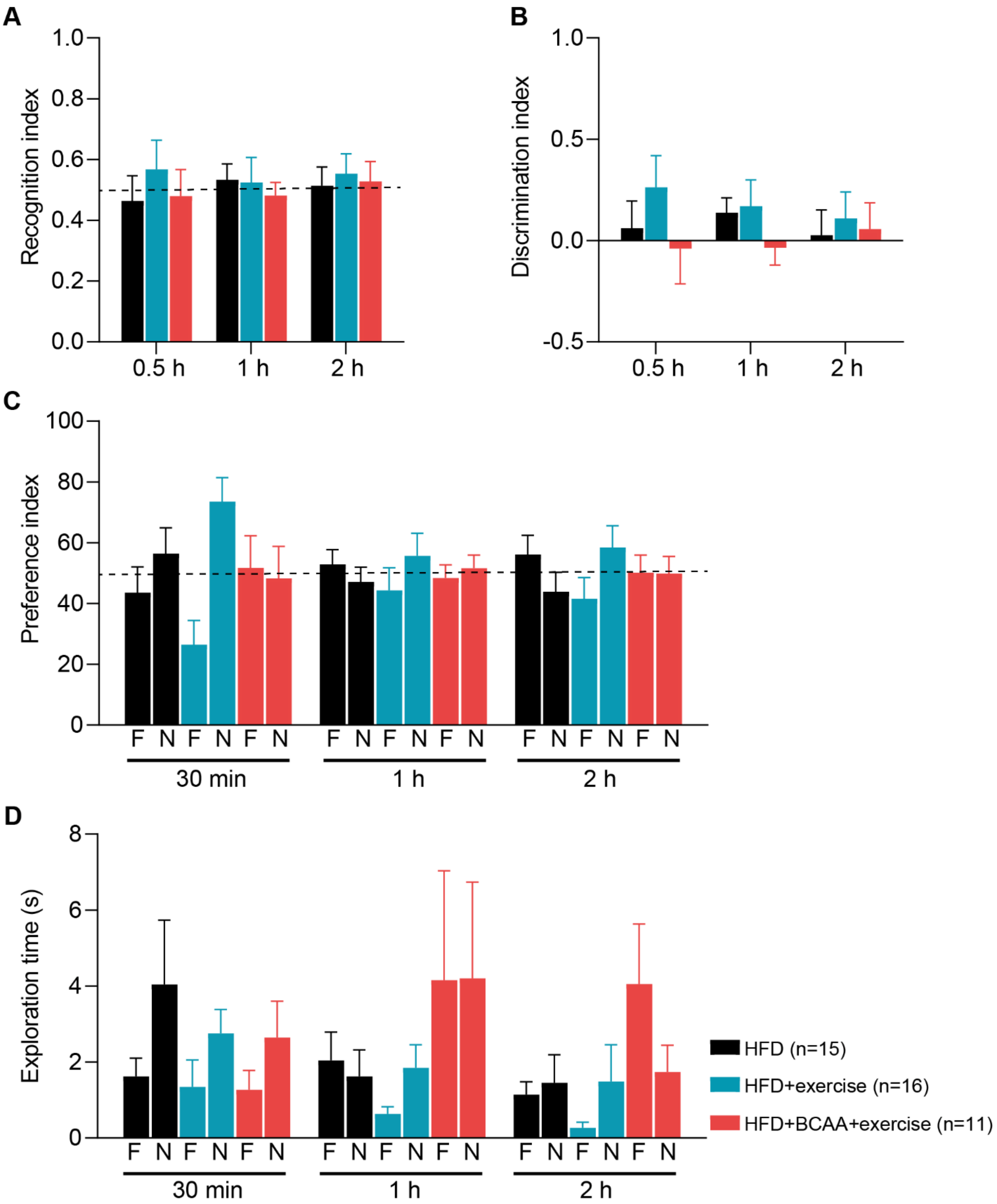

Supplementary Figure S11. Novel object recognition test. After 3 months of treatment, the novel object recognition test was performed and the (A) recognition index, (B) discrimination index, and (C) preference index and (D) exploration time around the familiar object (F) and the novel object (N) during the testing phase measured in the HFD, HFD+exercise, and HFD+BCAA+exercise group were calculated. No treatment effects were detected. Data are presented as mean ± SEM. High-fat diet (HFD), branched-chain amino acids (BCAA).

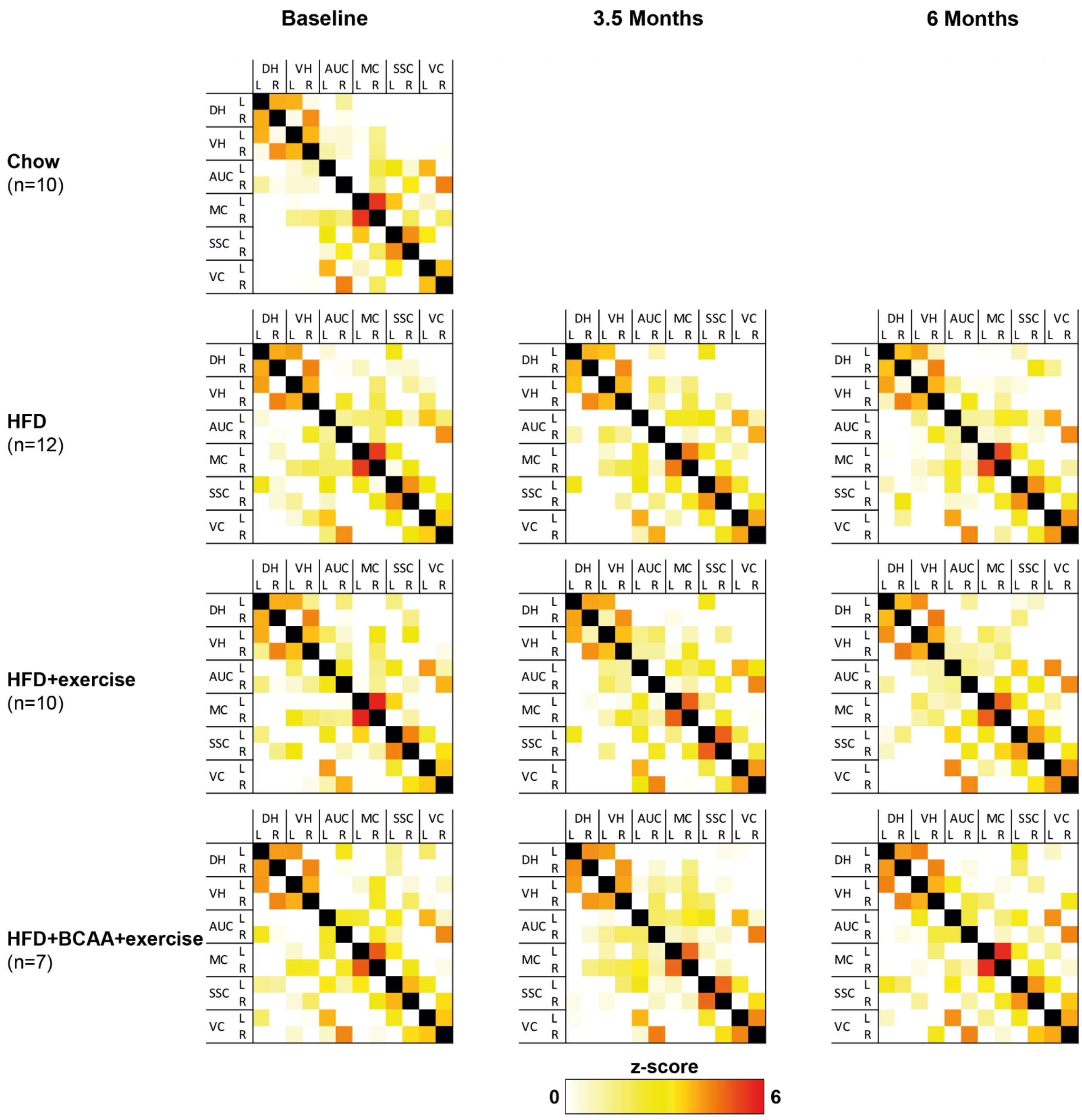

Supplementary Figure S12. Resting-state functional connectivity based on partial correlations. Functional connectivity between brain regions was measured by resting-state fMRI at baseline (chow) and after 3.5 and 6 months of treatment with HFD, HFD+exercise and HFD+BCAA+exercise. Partial correlation matrixes show the connectivity between the dorsal hippocampus (DH), ventral hippocampus (VH), auditory cortex (AC), motor cortex (MC), somatosensory cortex (SSC), and visual cortex (VC) for the left (L) and right (R) hemispheres, respectively at each neuroimaging time point. All significant results are summarized in the main article and summarized in Supplementary Table 12. High-fat diet (HFD), branched-chain amino acids (BCAA).

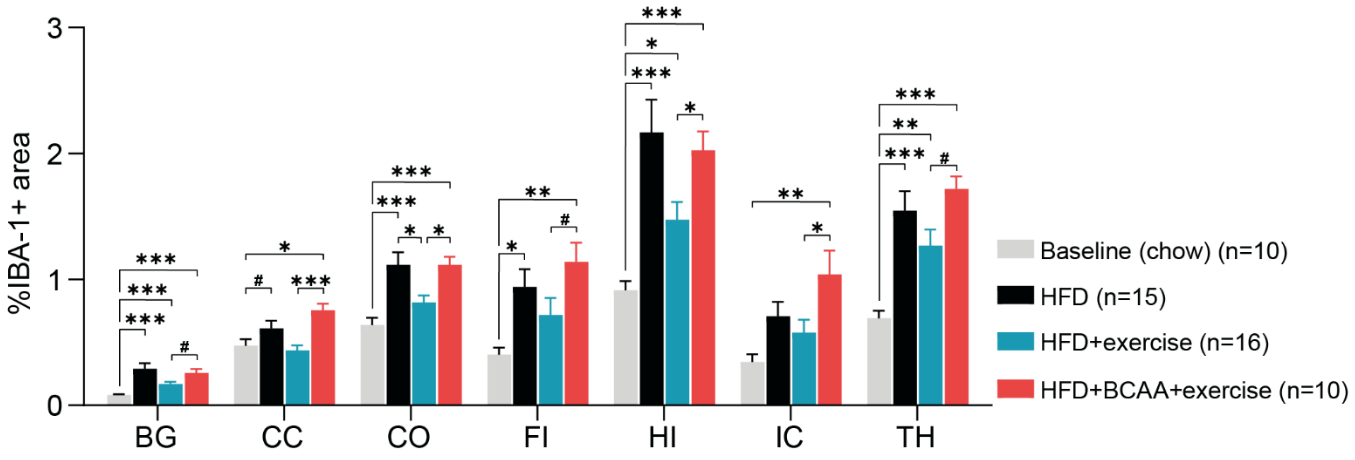

Supplementary Figure S13. Neuroinflammation. Immunohistochemical analysis of ionized calcium-binding adapter molecule (IBA-1) as measure of neuroinflammation in several brain regions of interest (basal ganglia (BG), corpus callosum (CC), cortex (C), fimbria (FI), hippocampus (HI), internal capsule (IC), thalamus (TH)). The IBA-1+ area was analyzed in young chow-fed animals (baseline), HFD, HFD+exercise, and HFD+BCAA+exercise groups. All HFD-fed groups showed a larger IBA-1+ area in the basal ganglia, hippocampus, and thalamus compared to young chow-fed mice. In addition, HFD and HFD+BCAA+exercise animals showed a larger IBA-1+ area in the corpus callosum, cortex, and fimbria compared to young chow-fed mice. In the internal capsule, HFD+BCAA+exercise animals had a larger IBA-1+ area than young chow-fed mice. Notably, exercise alone could counteract an HFD-induced enlargement of IBA-1+ area in the cortex. In contrast, HFD-BCAA+exercise mice showed significantly larger relative IBA-1+ area than exercising mice that did not receive BCAA supplementation in several ROI, including basal ganglia, corpus callosum, cortex, fimbria, hippocampus, internal capsule, and thalamus. High-fat diet (HFD), branched-chain amino acids (BCAA). Data are presented as mean ± SEM. * $p < 0.05$, ** $p < 0.01$, *** $p < 0.001$, # 0.05.

Supplementary Table S1. Ingredients and composition of special diets. High-fat diet (HFD, D12451 Research Diets, New Brunswick) and branched-chain amino acid enriched high-fat diet (HFD+BCAA, Research Diets, New Brunswick, USA). Percentages of macronutrients in the food are based on mass (gm %) and kilocalories (kcal %).

	HFD		HFD+BCAA	
	gm %	kcal %	gm %	kcal %
**Protein**	21	18	21	18
**Carbohydrates**	41	36	42	36
**Fat**	24	46	24	46
**Total**		100		100
**kcal/g**	4.6		4.7	
**Ingredients**	**gm**	**kcal**	**gm**	**kcal**
Casein	200	704	137.832	485
L-Alanine	0	0	0.139	1
L-Arginine	0	0	1.811	7
L-Asparagine	0	0	0.195	1
L-Aspartic Acid	0	0	0.139	1
L-Cysteine	3	12	3.333	13
L-Glutamine	0	0	0.473	2
L-Glutamic Acid	0	0	0.579	2
Glycine	0	0	0.083	0
L-Histidine	0	0	1.389	6
**L-Isoleucine**	**0**	**0**	**8.613**	**34**
**L-Leucine**	**0**	**0**	**17.475**	**70**
L-Lysine	0	0	3.928	16
DL-Methionine	0	0	1.513	6
L-Phenylalanine	0	0	2.539	10
L-Proline	0	0	0.486	2
L-Serine	0	0	0.278	1
L-Threonine	0	0	2.175	9
L-Tryptophan	0	0	0.603	2
L-Tyrosine	0	0	2.721	11
**L-Valine**	**0**	**0**	**9.159**	**37**
Corn starch	72.8	291	72.8	291
Maltodextrin-10	100	400	100	400
Sucrose	172.8	691	172.8	691
Cellulose	50	0	50	0
Soybean oil	25	225	25	225
Lard	177.5	1598	177.5	1598
Mineral mix S10026	10	0	10	0
Di-calcium phosphate	13	0	13	0
Calcium carbonate	5.5	0	5.5	0
Potassium citrate	16.5	0	16.5	0
Vitamin Mix V10001	10	40	10	40
Choline bitartrate	2	0	2	0

	gm/kg	%	gm/kg	%
Alanine	5.83	0.583	4.21	0.421
Arginine	6.99	0.699	6.99	0.699
Asparagine	8.16	0.816	5.9	0.59
Aspartic Acid	5.83	0.583	4.21	0.42
Cysteine	4.89	0.489	7.89	0.489
Glutamine	19.81	1.981	14.33	1.433
Glutamic Acid	24.24	2.424	17.53	1.753
Glycine	3.5	0.35	2.53	0.253
Histidine	5.36	0.536	5.36	0.536
**Isoleucine**	**8.62**	**0.862**	**16.12**	**1.612**
**Leucine**	**18.18**	**1.818**	**33.18**	**3.318**
Lysine	15.15	1.515	15.15	1.515
Methionine	5.83	0.583	5.83	0.583
Phenylalanine	9.79	0.979	9.79	0.979
Proline	20.51	2.051	14.83	1.483
Serine	11.65	1.165	8.43	0.843
Threonine	8.39	0.839	8.39	0.839
Tryptophan	2.33	0.233	2.33	0.233
Tyrosine	10.49	1.049	10.49	1.049
**Valine**	**10.72**	**1.072**	**18.22**	**1.822**

Supplementary Table S2. Imaging parameters.

	Anatomical T2*w	ASL	DTI	rsfMRI	T2w
**Imaging method**	GE	FAIR-ASL	4-shot spin-echo PI	Spin-echo EPI	T2 TurboRARE
**Echo time (ms)**	7.35	10.08	21	10	30
**Repetition time (s)**	0.86	12	7.75	1.8	2.5
**Image matrix (pixel x pixel)**	512 × 512	128 × 96	128 × 128	96 × 96	256 x 256
**Field-of-view (mm)**	40 × 40	25 × 25	20 × 20	25 × 25	20 x 20
**Spatial resolution (µm/pixel)**	78 × 78 × 500	195 × 260 × 1000	156 × 156 × 500	260 × 260 × 500	78 × 78 × 500
**Number of slices**	20 × 3	16	20	20	20
**Total acquisition time (min)**	8	13	35	11	4

Supplementary Table S3. Average area ± SEM of the regions of interest that were manually selected using the freehand tool in ImageJ group on the brain sections used for immunohistochemistry (IHC) and polarized light imaging (PLI). High-fat diet (HFD), branched-chain amino acids (BCAA).

Parameter	Bregma	ROI	Baseline chow		HFD		HFD+exercise		HFD+BCAA+exercise	
			Area (µm)	±SEM	Area (µm)	±SEM	Area (µm)	±SEM	Area (µm)	±SEM
IHC	0.5	Anterior commissure	151.87	35.04	233.98	57.32	144.74	39.88	487.54	154.17
		Basal ganglia	19746.19	4509.61	23384.39	4008.46	15816.18	3249.01	47150.49	14910.29
		Corpus callosum	647.61	99.39	574.43	75.39	491.09	58.00	672.81	212.76
		Cortex	27362.59	4289.74	28307.79	4379.68	20074.72	2242.99	44846.42	14181.68
		External capsule	693.08	177.67	581.32	108.12	387.85	87.10	821.95	259.92
	1.94	Corpus callosum	410.55	67.38	407.42	98.04	305.90	21.76	680.92	215.33
		Cortex	16793.07	2805.41	21043.36	2953.49	16253.70	2017.73	29194.26	9232.03
		External capsule	1159.82	189.62	887.26	120.53	884.44	122.26	1571.61	496.99
		Fimbria	1131.08	159.14	1436.19	131.39	1027.91	101.40	1392.53	440.36
		Hippocampus	62898.62	9829.14	73807.80	6070.54	65916.90	6299.24	90313.62	28559.68
		Internal capsule	1177.93	357.41	1283.61	176.59	969.14	257.27	1357.34	452.45
		Optic tract	396.84	74.25	518.29	51.97	452.02	80.84	443.98	140.40
		Thalamus	44237.45	5215.97	55673.12	14803.91	35466.59	4358.35	64849.18	20507.11
PLI	0.5	Anterior commissure	940.60	110.59	1011.78	78.16	1127.43	100.53	5729.58	1727.53
		Basal ganglia	22846.16	1578.46	22472.87	1242.40	22125.10	976.55	22795.57	6873.12
		Corpus callosum	1952.07	158.71	2345.47	200.41	1806.60	206.68	2155.41	649.88
		External capsule	847.85	69.89	935.18	50.81	1012.53	75.15	1052.22	317.26
		Motor cortex	5584.07	284.41	4897.53	307.22	4458.86	256.00	4273.50	1288.51
		Somatosensory cortex	17355.41	932.30	13563.87	830.41	14320.85	812.12	18200.21	5487.57
	0.14	Anterior commissure	4345.10	385.47	5549.01	406.47	4852.21	309.23	1052.29	317.28
		Basal ganglia	22940.74	866.56	23356.89	712.01	21447.89	1488.44	22290.14	6720.73
		Corpus callosum	1750.25	120.18	2005.34	114.83	1764.82	120.93	2607.18	786.09
		External capsule	1028.12	62.91	1069.82	50.08	1092.92	45.60	965.20	291.02
		Motor cortex	4650.52	353.45	4269.29	316.74	4287.57	420.08	4747.88	1431.54
		Somatosensory cortex	16425.19	999.11	17240.44	817.15	17375.15	556.73	16725.36	5042.88
		Fornix	2298.39	131.07	2148.33	157.18	1886.26	201.23	2686.88	849.67
	1.94	Corpus callosum	1706.53	187.14	2134.20	288.41	2622.86	226.99	2451.05	739.02
		Cortex	20523.80	1091.83	19825.40	786.39	19240.64	817.54	19225.38	5796.67
		External capsule	1857.21	218.35	2243.75	190.01	2651.50	130.04	2662.98	842.11

		**Fimbria**	1626.03	130.99	1561.07	129.97	1869.74	162.30	1443.35	435.19
		**Hippocampus**	1095.95	344.31	962.77	109.65	947.47	96.77	954.34	287.74
		**Internal capsule**	23785.64	1628.24	24136.57	1094.65	26013.48	1004.44	25360.74	7646.55
		**Optic tract**	924.55	138.22	1021.83	130.12	1197.82	111.23	1135.71	342.43

Supplementary Table S4. Sample size (number of mice or cages) per experiment. In total 4 animals of the HFD+BCAA+exercise group were excluded from analysis because they did not used the running wheel throughout the experiment. Furthermore, 2 mice died before the end of the experiment. The final group sample size was baseline(chow) n=10, HFD n=15, HFD+exercise n=16, HFD+BCAA+exercise n=11. Abbreviations: high-fat diet (HFD), branched-chain amino acids (BCAA), digital ventilated cages (DVC), reverse Morris Water Maze ((r)MWM), functional magnetic resonance imaging (fMRI), glucose transporter 1 (GLUT-1), ionized calcium-binding adapter molecule (IBA-1), doublecortin (DCX).

Parameter	Baseline (chow)	HFD	HFD+exercise	HFD+BCAA+exercise
**Body weight**	10	15	16	10
**Caloric intake (cage level)**	5	8	8	6
**Muscle weight**	9	15	16	10
**Fat depots and percentage**	8	15	16	10
**Glucose**	10	15	16	11
**Insulin**	10	14	15	11
**Cholesterol**	10	15	16	11
**Triglycerides**	10	15	16	11
**Serum amyloid A**	10	10	12	11
**E-selectin**	10	13	15	11
**Systolic blood pressure**	10	15	16	11
**DVC activity (cage level)**	6	8	8	6
**DVC running wheel distance (cage level)**			6	4
**Open field**	10	14	16	11
**Grip test**	10	15	16	11
**Rotarod**	10	15	16	11
**MWM - Acquisition**	10	15	16	11
**MWM - Probe**	10	15	16	11
**rMWM - Acquisition**		15	16	11
**rMWM - Probe (old platform location)**		15	16	10
**rMWM - Probe (new platform location)**		14	16	11
**Object recognition test**		15	16	11
**resting-state fMRI**	10	12	10	7
**Cerebral blood flow**	10	12	13	9
**Diffusion tensor imaging**	9	12	14	9
**Polarized light imaging**	9-10	14-15	14-16	10-11
**Cortical thickness**	10	15	15	11
**Hippocampal volume**	10	15	15	11
**GLUT-1 staining**	9-10	15	16	9-10
**IBA-1 staining**	10	15	16	10
**DCX staining**	10	15	16	11

Supplementary Table S5. Excluded mice per experiment and reason for exclusion. Abbreviations: high-fat diet (HFD), branched-chain amino acids (BCAA), digital ventilated cages (DVC), (reverse) Morris water maze ((r)MWM), functional magnetic resonance imaging (fMRI), glucose transporter 1 (GLUT-1), ionized calcium-binding adapter molecule (IBA-1), doublecortin (DCX), # = number of mice.

Parameter	Baseline (chow)	HFD	HFD+exercise	HFD+BCAA+exercise	Reason for exclusion
**Body weight**	0	0	0	1	statistical outlier (single-housed) (#1)
**Caloric intake (cage level)**	0	0	0	0	
**Muscle weight**	0	0	0	1	statistical outlier (#1)
**Fat depots and percentage**	2	0	0	1	missing measurements (#3)
**Glucose**	0	0	0	0	
**Insulin**	0	1	1	0	statistical outlier (#1)<br>missing measurements (#1)
**Cholesterol**	0	0	0	0	
**Triglycerides**	0	0	0	0	
**Serum amyloid A**	0	5	4	0	statistical outlier (#8)<br>missing measurement (#1)
**E-selectin**	0	2	1	0	statistical outlier (#2)<br>missing measurement (#1)
**Systolic blood pressure**	0	0	0	0	
**DVC activity (cage level)**	0	0	0	0	
**DVC distance (cage level)**			2	3	missing data due to technical problems (#4)<br>single house moused excluded (#1)
**Open field**	0	1	0	0	mouse was inactive (#1)
**Grip test**	0	0	0	0	
**Rotarod**	0	0	0	0	
**MWM - Acquisition**	0	0	0	0	
**MWM - Probe**	0	0	0	0	
**rMWM - Acquisition**		0	0	0	
**rMWM - Probe (old platform location)**		0	0	1	mouse was only floating in the water (#1)
**rMWM - Probe (new platform location)**		1	0	0	no video available due to technical problems (#1)
**Object recognition index**		0	0	0	
**Cerebral blood flow**	0	3	3	2	low body temperature (#8)
**resting-state fMRI**		3	6	4	very low CBF may influence functional connectivity (#8)<br>statistical outlier (#5)

**Diffusion tensor imaging**	1	3	2	2	bad realignment (#2)<br>movement resp. EPI related artifact (#5)<br>statistical outlier (#1)
**Polarized light imaging**	1	1	2	1	one or more regions of interest were not available (#5)
**Cortical thickness**	0	1	0	0	scan artifact (#1)
**Hippocampal volume**	0	1	0	0	scan artifact (#1)
**GLUT-1 staining**	1	0	0	2	one or more regions of interest were not available (#2)<br>technical issues (#1)
**IBA-1 staining**	0	0	0	1	one or more regions of interest were not available (#1)
**DCX staining**	0	0	0	0	

Supplementary Table S6. Summary statistics of body weight, caloric intake, muscle weight, and fat tissue analysis. Abbreviations: subcutaneous adipose tissue (sWAT), epididymal white adipose tissue (eWAT), mesenteric white adipose tissue (mWAT), high-fat diet (HFD), branched-chain amino acids (BCAA).

Parameters		Overall	HFD > Baseline (chow)		HFD+exercise > Baseline (chow)		HFD+BCAA+exercise > Baseline (chow)		HFD+exercise > HFD	HFD+BCAA+exercise < HFD	HFD+BCAA+exercise < HFD+exercise
			p-value	relative change (%)	p-value	relative change (%)	p-value	relative change (%)	p-value	p-value	p-value
**Body weight**	**Baseline**	F(3,47)=2.406, p<0.079									
	**Month 1**	F(2,38)=6.833, p<0.003								p<0.002	p<0.079
	**Month 2**	F(2,38)=3.889, p<0.029								p<0.029	p<0.075
	**Month 3**	F(2,38)=8.050, p<0.001								p<0.003	p<0.001
	**Month 4**	F(2,38)=6.375, p<0.004								p<0.013	p<0.005
	**Month 5**	F(2,38)=6.868, p<0.003								p<0.014	p<0.003
	**Month 6**	F(2,38)=10.373, p<0.001								p<0.002	p<0.001
**Caloric intake**	**Baseline**	F(3,23)=1.122, p<0.361									
**Muscle weight**	**Gastrocnemius**	F(3,46)=12.015, p<0.001	p<0.011	113.7	p<0.001	124.7	p<0.010	114.9	p<0.018		
	**Soleus**	F(3,46)=19.813, p<0.001	p<0.001	139.1	p<0.001	168.8	p<0.001	153.9	p<0.007		
	**Quadriceps**	F(3,46)=2.086, p<0.115									
**Fat**	**sWAT weight**	F(3,45)=30.228, p<0.001	p<0.001	846.1	p<0.001	863.7	p<0.001	630.7			
	**eWAT weight**	F(3,45)=26.538, p<0.001	p<0.001	518.7	p<0.001	494.3	p<0.001	557.0			
	**mWAT weight**	F(3,45)=23.845, p<0.001	p<0.001	504.0	p<0.001	538.2	p<0.007	355.9			p<0.062
	**Fat percentage**	F(3,45)=44.878, p<0.001	p<0.001	353.0	p<0.001	347.1	p<0.001	346.7			
		**Overall**	**HFD < Baseline (chow)**		**HFD+exercise < Baseline (chow)**		**HFD+BCAA+exercise < Baseline (chow)**		**HFD+exercise > HFD**	**HFD+BCAA+exercise > HFD**	**HFD+BCAA+exercise > HFD+exercise**
**Muscle (% total BW)**	**Gastrocnemius**	F(3,46)=27.978, p<0.001	p<0.001	67.3	p<0.001	71.7	p<0.001	113.0		p<0.003	p<0.062
	**Soleus**	F(3,46)=5.071, p<0.004	p<0.002	82.5					p<0.078	p<0.054	
	**Quadriceps**	F(3,46)=10.985, p<0.001	p<0.007	66.8	p<0.009	67.9					
		**Overall**	**HFD > Baseline (chow)**		**HFD+exercise > Baseline (chow)**		**HFD+BCAA+exercise > Baseline (chow)**		**HFD+exercise > HFD**	**HFD+BCAA+exercise < HFD**	**HFD+BCAA+exercise > HFD+exercise**
**Fat (% total BW)**	**sWAT**	F(3,45)=36.672, p<0.001	p<0.001	482.2	p<0.001	483.9	p<0.001	86.5			
	**eWAT**	F(3,45)=20.996, p<0.001	p<0.001	305.4	p<0.001	280.7	p<0.001	133.1			p<0.02
	**mWAT**	F(3,45)=21.214, p<0.001	p<0.001	286.9	p<0.001	300.2	p<0.008	77.7			

Parameter	Effects	Baseline → 1 months		1 months → 6 months	Baseline → 6 months	
		p-value	relative change (%)	p-value	p-value	relative change (%)
**Body weight**	Time effect all groups	↑F(1,38)=158.324, p<0.001	122.3	↑F(5,190)=270.208, p<0.001	↑F(1,38)=745.986, p<0.001	177.0
	HFD+BCAA+exercise < HFD	p<0.003		p<0.004	p<0.004	
	HFD+BCAA+exercise < HFD-exercise			p<0.002	p<0.002	
**Caloric intake**	Time effect all groups	↑F(1,19)=45.637, p<0.001	116.0	↓F(5,95)=13.358, p<0.001	↑ F(1,19)=8.637, p<0.008	106.3

Supplementary Table S7. Summary statistics of fasting blood and plasma markers and systolic blood pressure analysis. Abbreviations: high-fat diet (HFD), branched-chain amino acids (BCAA).

Parameters			Overall	HFD > Baseline (chow)	HFD+RW > Baseline (chow)	HFD+BCAA+RW > Baseline (chow)	HFD+RW > HFD	HFD+BCAA+RW < HFD	HFD+BCAA+RW < HFD+RW
**Fasting blood**	**Glucose**	Baseline	F(3,48)=0.681; p<0.568						
**Fasting Plasma**	**Insulin**	Baseline	F(3,46)=0.963; p<0.418						
		3 Months	F(2,37)=3.609; p<0.037						p<0.031
		6 Months	F(2,37)=6.134; p<0.005					p<0.047	p<0.004
	**Cholesterol**	Baseline	F(3,48)=0.306; p<0.821						
	**Triglycerides**	Baseline	F(3,48)=0.081; p<0.970						
	**Serum amyloid A**	Baseline	F(3,39)=3.909; p<0.016			p<0.034			p<0.042
	**E-selectin**	Baseline	F(3,45)=0.283; p<0.837						
**Systolic blood pressure**		Baseline	F(3,48)=0.630; p<0.599						

Parameter		Effects	Baseline → 3 months		3 months → 6 months	Baseline → 6 months	
			p-value	relative change (%)	p-value	p-value	relative change (%)
**Blood plasma**	**Glucose**	Time effect all groups	↑F(1,39)=33.431, p<0.001	125.7	↓F(1,39)=3.708, p<0.061	↑F(1,39)=17.338, p<0.001	114.3
	**Insulin**	Time effect all groups	↑F(1,37)=165.843, p<0.001	659.0	↑F(1,37)=8.335, p<0.006	↑F(1,37)=161.119, p<0.001	1034.5
		HFD+BCAA+exercise < HFD				p<0.061	
		HFD+BCAA+exercise < HFD+exercise			p<0.004	p<0.023	
	**Cholesterol**	Time effect all groups	↑F(1,39)=36.227, p<0.001	162.7	↑F(1,39)=376.410, p<0.001	↑F(1,39)=476.049, p<0.001	425.1
	**Triglycerides**	Time effect all groups	↑F(1,39)=18.863, p<0.001	178.1	↑F(1,39)=149.310, p<0.001	↑F(1,39)=254.231, p<0.001	437.4
	**SAA**	Time effect all groups	↑F(1,30)=10.610, p<0.003	200.4	↑F(1,30)=11.062, p<0.002	↑F(1,30)=16.280, p<0.001	265.6
	**E-selectin**	Time effect all groups	↑F(1,36)=7.659, p<0.009	113.3	↓F(1,36)=4.835, p<0.034		
			**Baseline → 2 months**		**2 months → 6 months**	**Baseline → 6 months**	
**Systolic blood pressure**		Time effect all groups	↑F(1,39)=17.360, p<0.001	115.8	↑F(2,78)=6.292, p<0.003	↑F(1,39)=49.525, p<0.001	125.0

Supplementary Table S8. Summary statistics of DVC metrics and open field parameters. Abbreviations: digital ventilated cages (DVC), high-fat diet (HFD), branched-chain amino acids (BCAA).

Parameters			Overall	HFD > Baseline (chow)	HFD+RW > Baseline (chow)	HFD+BCAA+RW > Baseline (chow)	HFD+RW > HFD	HFD+BCAA+RW < HFD	HFD+BCAA+RW > HFD+RW
DVC	Activity daytime	Baseline	F(3,24)=0.044, p<0.987						
		Month 6	F(2,19)=3.19, p<0.004					p<0.003	
				**HFD > Baseline (chow)**	**HFD+RW > Baseline (chow)**	**HFD+BCAA+RW > Baseline (chow)**	**HFD+RW > HFD**	**HFD+BCAA+RW > HFD**	**HFD+BCAA+RW > HFD+RW**
	Activity nighttime	Baseline	F(3,24)=0.588, p<0.629						
		Month 1	F(2,19)=1.751, p<0.200						
		Month 2	F(2,19)=2.054, p<0.156						
		Month 3	F(2,19)=2.566, p<0.103						
		Month 4	F(2,19)=5.838, p<0.011					p<0.01	p<0.042
		Month 5	F(2,19)=0.620, p<0.548						
		Month 6	F(2,19)=3.060, p<0.070						
Open field	Distance	Baseline	F(3,47)=1.399, p<0.255						
	Velocity	Baseline	F(3,47)=1.428, p<0.246						
				**HFD > Baseline (chow)**	**HFD+RW > Baseline (chow)**	**HFD+BCAA+RW > Baseline (chow)**	**HFD+RW > HFD**	**HFD+BCAA+RW < HFD**	**HFD+BCAA+RW < HFD+RW**
	Center (frequency)	Baseline	F(3,47)=3.825, p<0.016						p<0.010
	Corner (frequency)	Baseline	F(3,47)=1.659, p<0.189						
	Periphery (frequency)	Baseline	F(3,47)=2.216,p<0.099						
	Center (time)	Baseline	F(3,47)=3.344, p<0.027					p<0.030	p<0.049
	Periphery (time)	Baseline	F(3,47)=3.198, p<0.032					p<0.064	p<0.028
				**HFD > Baseline (chow)**	**HFD+RW > Baseline (chow)**	**HFD+BCAA+RW > Baseline (chow)**	**HFD+RW > HFD**	**HFD+BCAA+RW < HFD**	**HFD+BCAA+RW < HFD+RW**
	Corner (time)	Baseline	F(3,47)=5.924, p<0.002			p<0.063		p<0.003	p<0.002

Parameter		Effects	Baseline → 1 months		1 month → 6 months	Baseline → 6 months	
			p-value	relative change (%)	p-value	p-value	relative change (%)
DVC	Activity - daytime	Time effect all groups	↑F(1,19)=5.216, p<0.034	171.8	↑F(5,95)=9.237, p<0.001		
		Time effect HFD group				↑F(1,7)=8.528, p<0.022	147.6
		Time effect HFD+RW group				↑F(1,7)=4.865, p<0.063	128.6
		HFD+BCAA+exercise < HFD			p<0.048	p<0.005	
	Activity - nighttime	Time effect all groups	↑F(1,19)=18.943, p<0.001	188.4			
		Time effect HFD+BCAA+RW group			↓F(5,25)=15.582, p<0.001		
			**1 months → 6 months**				
DVC	Distance - daytime	Time effect all groups	↓F(5,40)=13.078, p<0.001	38.7			
	Distance - nighttime	Time effect all groups	↓F(5,40)=23.144, p<0.001	21.8			
			**Baseline → 5 months**				
Open field	Distance	Time effect all groups	↓F(1,39)=22.467, p<0.001	81.0			
		HFD+BCAA+exercise < HFD+exercise	p<0.033				
	Velocity	Time effect all groups	↓F(1,38)=22.584, p<0.001	81.3			
		HFD+BCAA+exercise < HFD+exercise	p<0.031				
	Center (time)	Time effect all groups					
	Corner (time)	Time effect all groups	↑F(1,38)=9.072, p<0.005	111.9			
	Periphery (time)	Time effect all groups					
	Center (frequency)	Time effect all groups					
	Corner (frequency)	Time effect all groups	↓F(1,38)=12.710, p<0.001	72.5			
		HFD+BCAA+exercise < HFD+exercise	p<0.006				
	Periphery (frequency)	Time effect all groups	↓F(1,38)=14.606, p<0.001	69.4			
		HFD+BCAA+exercise < HFD+exercise	p<0.011				

Supplementary Table S9. Summary statistics of the grip strength test and Rotarod. Abbreviations: high-fat diet (HFD), branched-chain amino acids (BCAA).

Parameters			Overall	HFD > Baseline (chow)	HFD+exercise > Baseline (chow)	HFD+BCAA+exercise > Baseline (chow)	HFD+exercise > HFD	HFD+BCAA+exercise > HFD	HFD+BCAA+exercise > HFD+exercise
**Grip test - 2 Paws**	**Total force**	**Baseline**	F(3,48)=1.130, p<0.347						
		**Month 1**	F(2,39)=6.377, p<0.004					p<0.006	p<0.012
		**Month 5**	F(2,39)=0.962, p<0.391						
	**Peak force**	**Baseline**	F(3,48)=0.693, p<0.561						
**Grip test - 4 Paws**	**Total force**	**Baseline**	F(3,48)=0.719, p<0.546						
		**Month 1**	F(2,39)=3.781, p<0.032					p<0.038	p<0.065
		**Month 5**	F(2,39)=1.047, p<0.361						
	**Peak force**	**Baseline**	F(3,48)=0.916, p<0.440						
**Rotarod**	**Latency to fall**	**Baseline**	F(3,48)=1.159, p<0.335						

Parameter		Effects	Baseline → 1 month		1 month → 5 months	Baseline → 5 months	
			**p-value**	**relative change (%)**	**p-value**	**p-value**	**relative change (%)**
**Grip test - 2 Paws**	**Total force**	Time effect all groups	↑F(1,39)=7.952, p<0.008	112.4		↓F(1,39)=6.855, p<0.013	85.1
		Time effect HFD+exercise group			↓F(1,15)=5.513, p<0.033		
		Time effect HFD+BCAA+exercise group			↓F(1,10)=56.767, p<0.001		
		HFD+BCAA+exercise > HFD	p<0.055				
		HFD+BCAA+exercise > HFD+exercise	p<0.015				
	**Peak force**	Time effect all groups			↓F(1,39)=15.765, p<0.001	↓F(1,39)=3.961, p<0.054	92.9
**Grip test - 4 Paws**	**Total force**	Time effect all groups	↑F(1,39)=27.287, p<0.001	131.8		↑F(1,39)=36.558, p<0.001	133.0
		Time effect HFD+BCAA+exercise group			↓F(1,10)=9.548, p<0.011		
		HFD+BCAA+exercise > HFD	p<0.049				
		HFD+BCAA+exercise > HFD+exercise	p<0.073				
	**Peak force**	Time effect all groups	↓F(1,39)=4.274, p<0.045	92.3		↓F(1,39)=26.076, p<0.001	89.0
**Rotarod**	**Latency to fall**	Time effect all groups	↓F(1,39)=27.757, p<0.001	72.2	↓F(1,39)=96.396, p<0.001	↓F(1,39)=128.777, p<0.001	39.6

Supplementary Table S10. Summary statistics of the (reverse) Morris Water Maze. Abbreviations: (reverse) Morris water maze ((r)MWM), high-fat diet (HFD), branched-chain amino acids (BCAA).

**Parameter**		**Effects**	**Acquisition day 1 → Acquisition day 4**	
			**p-value**	**relative change (%)**
**Morris water maze - baseline**	**Escape latency**	Time effect all groups	↓F(3,144)=4.311, p<0.006	85.7
	**Distance**	Time effect all groups	↓F(3,144)=32.280, p<0.001	59.5
	**Velocity**	Time effect all groups	↓F(3,144)=44.186, p<0.001	74.1
			**Acquisition day 1 → Acquisition day 2**	
**reverse Morris water maze - month 5**	**Escape latency**	Time effect all groups	↓F(1,39)=6.654, p<0.014	80.1
		HFD+BCAA+exercise < HFD	p<0.061	
	**Distance**	Time effect all groups	↓F(1,39)=9.872, p<0.003	75.5
	**Velocity**	Time effect all groups	↓F(1,39)=16.824, p<0.001	91.0

**Parameters**		**Overall**	**HFD > Baseline (chow)**	**HFD+exercise > Baseline (chow)**	**HFD+BCAA+exercise > Baseline (chow)**	**HFD+exercise > HFD**	**HFD+BCAA+exercise > HFD**	**HFD+BCAA+exercise > HFD+exercise**
**MWM - Probe**	**Platform location (time)**	χ2(3)=7.154, p<0.067						
	**Platform (frequency)**	χ2(3)=7.476, p<0.058						
	**Platform quadrant (time)**	F(3,48)=1.592, p<0.204						
	**Platform quadrant (frequency)**	F(3,48)=2.722, p<0.055						
**rMWM- Probe Day1**	**Platform location (time)**	χ2(2)=0.396, p<0.820						
	**Platform (frequency)**	χ2(2)=0.807, p<0.807						
	**Platform quadrant (time)**	F(2,38)=0.055, p<0.946						
	**Platform quadrant (frequency)**	F(2,38)=0.967, p<0.390						
**rMWM - Probe Day 2**	**Platform location (time)**	F(2,38)=1.392, p<0.261						
	**Platform (frequency)**	χ2(2)=1.578, p<0.454						
	**Platform quadrant (time)**	F(2,38)=0.693, p<0.506						
	**Platform quadrant (frequency)**	F(2,38)=4.098, p<0.024					p<0.025	

Supplementary Table S11. Summary statistics of the resting-state functional connectivity analysis based on total correlations. Abbreviations: high-fat diet (HFD), branched-chain amino acids (BCAA), left (L), right (R).

**Connection**			**Effects**	**Baseline → 3.5 months**	**3.5 months → 6 months**	**Baseline → 6 months**
**Intrahemispheric**	**Auditory cortex L**	**Ventral hippocampus L**	Time effect all groups	↓F(1,26)=6.694, p<0.016		↓F(1,26)=13.294, p<0.001
	**Auditory cortex L**	**Dorsal hippocampus L**	Time effect all groups			↓F(1,26)=6.484, p<0.017
	**Auditory cortex R**	**Dorsal hippocampus R**	Time effect all groups	↓F(1,26)=3.985, p<0.056		
	**Motor cortex R**	**Ventral hippocampus R**	Time effect all groups		↓F(1,26)=5.061, p<0.033	
	**Somatosensory cortex L**	**Ventral hippocampus L**	Time effect all groups			↓F(1,26)=7.715, p<0.01
	**Somatosensory cortex L**	**Auditory cortex L**	Time effect all groups	↓F(1,26)=21.238, p<0.025		↓F(1,26)=5.772, p<0.024
	**Visual cortex L**	**Ventral hippocampus L**	Time effect all groups	↓F(1,26)=8.064, p<0.009		↓F(1,26)=19.112, p<0.001
	**Visual cortex L**	**Auditory cortex L**	Time effect all groups	↓F(1,26)=6.351, p<0.018		↓F(1,26)=5.785, p<0.024
	**Visual cortex L**	**Motor cortex L**	Time effect all groups			↓F(1,26)=4.038, p<0.055
	**Visual cortex L**	**Somatosensory cortex L**	Time effect all groups	↓F(1,26)=6.983, p<0.014		↓F(1,26)=9.52, p<0.005
	**Visual cortex L**	**Dorsal hippocampus L**	Time effect all groups	↓F(1,26)=4.119, p<0.053		↓F(1,26)=7.506, p<0.011
	**Visual cortex R**	**Dorsal hippocampus R**	Time effect all groups	↓F(1,26)=5.205, p<0.031		
	**Visual cortex R**	**Ventral hippocampus R**	Time effect all groups	↓F(1,26)=7.022, p<0.014		↓F(1,26)=5.6, p<0.026
	**Visual cortex R**	**Auditory cortex R**	Time effect all groups	↓F(1,26)=5.121, p<0.032		
	**Visual cortex R**	**Motor cortex R**	Time effect all groups	↓F(1,26)=1.15, p<0.037		
	**Visual cortex R**	**Somatosensory cortex R**	Time effect all groups	↓F(1,26)=4.133, p<0.052		
	**Motor cortex L**	**Dorsal hippocampus L**	HFD+BCAA+exercise < HFD			p<0.041
			HFD+BCAA+exercise < HFD+exercise			p<0.054
**Interhemispheric**	**Auditory cortex L**	**Dorsal hippocampus R**	Time effect all groups	↓F(1,26)=4.991, p<0.034		↓F(1,26)=6.335, p<0.018
	**Auditory cortex L**	**Ventral hippocampus R**	Time effect all groups	↓F(1,26)=5.662, p<0.025		↓F(1,26)=16.852, p<0.001
	**Auditory cortex R**	**Auditory cortex L**	Time effect all groups	↓F(1,26)=9.673, p<0.004		↓F(1,26)=9.621, p<0.005
	**Motor cortex R**	**Ventral hippocampus L**	Time effect all groups		↓F(1,26)=5.127, p<0.032	
	**Somatosensory cortex L**	**Ventral hippocampus R**	Time effect all groups			↓F(1,26)=8.719, p<0.007
	**Somatosensory cortex L**	**Auditory cortex R**	Time effect all groups			↓F(1,26)=4.75, p<0.039
	**Somatosensory cortex L**	**Motor cortex R**	Time effect all groups			↓F(1,26)=3.44, p<0.075
	**Somatosensory cortex R**	**Auditory cortex L**	Time effect all groups			↓F(1,26)=4.165, p<0.052
	**Somatosensory cortex R**	**Somatosensory cortex L**	Time effect all groups			↓F(1,26)=4.595, p<0.042
	**Visual cortex L**	**Dorsal hippocampus R**	Time effect all groups	↓F(1,26)=6.01, p<0.021		↓F(1,26)=8.402, p<0.008
	**Visual cortex L**	**Ventral hippocampus R**	Time effect all groups	↓F(1,26)=8.202, p<0.008		↓F(1,26)=24.554, p<0.001
	**Visual cortex L**	**Auditory cortex R**	Time effect all groups	↓F(1,26)=4.393, p<0.046		↓F(1,26)=34.008, p<0.008
	**Visual cortex L**	**Motor cortex R**	Time effect all groups			↓F(1,26)=6.108, p<0.02
	**Visual cortex L**	**Somatosensory cortex R**	Time effect all groups			↓F(1,26)=5.424, p<0.028
	**Visual cortex R**	**Dorsal hippocampus L**	Time effect all groups	↓F(1,26)=4.188, p<0.051		
	**Visual cortex R**	**Ventral hippocampus L**	Time effect all groups	↓F(1,26)=8.815, p<0.006		↓F(1,26)=6.46, p<0.017
	**Visual cortex R**	**Auditory cortex L**	Time effect all groups	↓F(1,26)=8.189, p<0.008		↓F(1,26)=7.07, p<0.013
	**Visual cortex R**	**Motor cortex L**	Time effect all groups	↓F(1,26)=3.667, p<0.067		
	**Visual cortex R**	**Somatosensory cortex L**	Time effect all groups	↓F(1,26)=7.681, p<0.01		↓F(1,26)=3.895, p<0.059

	**Visual cortex R**	**Visual cortex L**	Time effect all groups			↓F(1,26)=4.686, p<0.04
	**Ventral hippocampus R**	**Dorsal hippocampus L**	HFD+BCAA+exercise < HFD			p<0.03
			HFD+BCAA+exercise < HFD+exercise			p<0.034
	**Motor cortex L**	**Auditory cortex R**	HFD+BCAA+exercise < HFD			p<0.051
			HFD+BCAA+exercise < HFD+exercise			p<0.027

Supplementary Table S12. Summary statistics of the resting-state functional connectivity analysis based on partial correlations. Abbreviations: high-fat diet (HFD), branched-chain amino acids (BCAA), left (L), right (R).

Connection			Effects	Baseline → 3.5 months	3.5 months → 6 months	Baseline → 6 months
Intrahemispheric	**Auditory cortex L**	**Ventral hippocampus L**	Time effect all groups			↑F(1,26)=7.006, p<0.014
	**Motor cortex L**	**Dorsal hippocampus L**	Time effect all groups			↑F(1,26)=4.541, p<0.043
	**Motor cortex L**	**Auditory cortex L**	Time effect all groups	↑F(1,26)=3.6, p<0.069	↓F(1,26)=6.578, p<0.016	
	**Motor cortex R**	**Dorsal hippocampus R**	Time effect all groups	↑F(1,26)=14.281, p<0.001	↑F(1,26)=3.912, p<0.059	
	**Somatosensory cortex L**	**Motor cortex L**	Time effect all groups		↑F(1,26)=4.803, p<0.038	
	**Somatosensory cortex R**	**Auditory cortex R**	Time effect all groups	↑F(1,26)=5.316, p<0.029		↑F(1,26)=4.12, p<0.053
	**Ventral hippocampus L**	**Dorsal hippocampus L**	Time effect all groups		↑F(1,26)=4.802, p<0.038	
	**Visual cortex L**	**Auditory cortex L**	Time effect all groups		↑F(1,26)=6.262, p<0.019	↑F(1,26)=3.548, p<0.071
	**Auditory cortex L**	**Ventral hippocampus L**	Time effect HFD group			↑F(1,11)=12, p<0.005
			Time effect HFD+BCAA+exercise group			↑F(1,6)=8, p<0.03
			HFD+exercise < HFD	p<0.027		
	**Motor cortex L**	**Auditory cortex L**	Time effect HFD+exercise group	↑F(1,9)=12.204, p<0.007		
			Time effect HFD+BCAA+exercise group			↓F(1,6)=7.169, p<0.037
	**Somatosensory cortex L**	**Motor cortex L**	Time effect HFD+exercise group	↓F(1,9)=28.593, p<0.001		
			Time effect HFD+BCAA+exercise group			
	**Somatosensory cortex R**	**Ventral hippocampus R**	Time effect HFD group	↑F(1,11)=4.448 p<0.035	↓F(1,11)=4.448 p<0.031	
			Time effect HFD+BCAA+exercise group	↓F(1,6)=9.801 p<0.02		
Interhemispheric	**Auditory cortex R**	**Dorsal hippocampus L**	Time effect all groups	↑F(1,26)=3.960, p<0.057		
	**Auditory cortex R**	**Ventral hippocampus L**	Time effect all groups	↑F(1,26)=13.091, p<0.001		
	**Auditory cortex R**	**Auditory cortex L**	Time effect all groups	↓F(1,26)=6.374, p<0.018		
	**Motor cortex L**	**Ventral hippocampus R**	Time effect all groups			↑F(1,26)=5.704, p<0.024
	**Motor cortex R**	**Auditory cortex L**	Time effect all groups		↑F(1,26)=3.39, p<0.077	
			HFD+exercise < HFD		p<0.002	p<0.029
			HFD+BCAA+exercise < HFD			p<0.006
			HFD+exercise < HFD+BCAA+exercise		p<0.065	
	**Motor cortex R**	**Motor cortex L**	Time effect all groups	↓F(1,26)=16.162, p<0.014		
	**Somatosensory cortex L**	**Dorsal hippocampus R**	Time effect all groups		↑F(1,26)=7.104, p<0.013	
	**Somatosensory cortex R**	**Dorsal hippocampus L**	Time effect all groups	↑F(1,26)=3.476, p<0.074		↑F(1,26)=3.531, p<0.072
	**Somatosensory cortex R**	**Somatosensory cortex L**	Time effect all groups	↑F(1,26)=3.486, p<0.073		
	**Visual cortex L**	**Ventral hippocampus R**	Time effect all groups	↑F(1,26)=3.931, p<0.058		↓F(1,26)=8.008, p<0.009
	**Visual cortex R**	**Ventral hippocampus L**	Time effect all groups	↑F(1,26)=3.308, p<0.08		

	**Visual cortex R**	**Auditory cortex L**	Time effect all groups		↓$F(1,26)=5.155$, $p<0.032$	
	**Visual cortex R**	**Visual cortex L**	Time effect all groups	↑$F(1,26)=10.783$, $p<0.003$		↑$F(1,26)=4.636$, $p<0.041$
	**Motor cortex R**	**Auditory cortex L**	Time effect HFD+BCAA+exercise group	↑$F(1,6)=22.859$ $p<0.003$		
	**Ventral hippocampus R**	**Dorsal hippocampus L**	HFD+exercise > HFD			$p<0.07$
			HFD+BCAA+exercise < HFD+exercise			$p<0.014$
	**Somatosensory cortex L**	**Motor cortex R**	HFD+exercise < HFD			$p<0.026$
	**Visual cortex L**	**Dorsal hippocampus L**	HFD+BCAA+exercise > HFD+exercise			$p<0.034$

Supplementary Table S13. Summary statistics of cerebral blood flow analysis. Abbreviations: high-fat diet (HFD), branched-chain amino acids (BCAA).

Parameter		Effects	Baseline → 1 months	1 months → 6 months	Baseline → 6 months	
			p-value	p-value	p-value	relative change (%)
**Vasodilation**	**Cortex**	**Time effect all groups**		↓F(1,31)=4.823, p<0.036	↓F(1,31)=5.682, p<0.023	89.7
	**Hippocampus**	**Time effect all groups**		↓F(1,31)=9.177, p<0.005		
		**Time HFD+exercise group**			↓F(1,12)=6.737, p<0.023	72.0
		**Time HFD+BCAA+exercise group**			↓F(1,8)=9.568, p<0.015	70.6
		**HFD+exercise < HFD**		p<0.047		
	**Thalamus**	**Time effect all groups**		↓F(1,31)=9.741, p<0.004	↓F(1,31)=7.104, p<0.012	85.0
**Vasoconstriction**	**Cortex**	**Time effect all groups**				
		**Time HFD+exercise group**			↓F(1,12)=7.178, p<0.020	75.7
	**Hippocampus**	**Time effect all groups**		↓F(1,31)=5.757, p<0.023		
		**Time HFD+exercise group**			↓F(1,12)=9.692, p<0.009	70.1
	**Thalamus**	**Time effect all groups**		↓F(1,31)=5.322, p<0.028	↓F(1,31)=3.572, p<0.068	92.24
**Vasoreactivity**	**Cortex**	**Time effect all groups**				
	**Hippocampus**	**Time effect all groups**				
	**Thalamus**	**Time effect all groups**				

Parameters			Overall	HFD < Baseline (chow)	HFD+RW > Baseline (chow)	HFD+BCAA+RW < Baseline (chow)	HFD+RW < HFD	HFD+BCAA+RW < HFD	HFD+BCAA+RW > HFD+RW
**Vasodilation**	**Cortex**	**Baseline**	F(3,40)=1.834, p<0.157						
		**Month 6**	F(2,31)=1.622, p<0.214						
	**Hippocampus**	**Baseline**	F(3,40)=1.660, p<0.191						
		**Month 6**	F(2,31)=4.039, p<0.028				p<0.077	p<0.038	
	**Thalamus**	**Baseline**	F(3,40)=0.714, p<0.550						
**Vasoconstriction**	**Cortex**	**Baseline**	F(3,40)=3.919, p<0.015				p<0.008		
		**Month 6**	F(2,31)=3.365, p<0.048						
	**Hippocampus**	**Baseline**	F(3,40)=1.926, p<0.141						
		**Month 6**	F(2,31)=1.386, p<0.265						
	**Thalamus**	**Baseline**	F(3,40)=1.171, p<0.333						
**Vasoreactivity**	**Cortex**	**Baseline**	F(3,40)=6.445, p<0.001	p<0.003		p<0.026	p<0.019		
	**Hippocampus**	**Baseline**	F(3,40)=3.111, p<0.037	p<0.022					
	**Thalamus**	**Baseline**	F(3,40)=1.299, p<0.288						

Supplementary Table S14. Summary statistics of immunohistochemical analysis of glucose transporter 1 (GLUT-1). Abbreviations: high-fat diet (HFD), branched-chain amino acids (BCAA).

Parameters		Overall	HFD < Baseline (chow)	HFD+exercise < Baseline (chow)	HFD+BCAA+exercise > Baseline (chow)	HFD+exercise > HFD	HFD+BCAA+exercise > HFD	HFD+BCAA+exercise > HFD+exercise
**relative GLUT-1+ area**	**Anterior commissure**	F(3,45)=2.791, p<0.051						
	**Basal ganglia**	$\chi^2(3)$=13.024, p<0.005						p<0.003
	**Corpus callosum**	F(3,45)=2.679, p<0.058						
	**Cortex**	F(3,45)=7.035, p<0.001			p<0.004		p<0.029	p<0.001
	**External capsule**	F(3,45)=3.160, p<0.034						p<0.046
	**Fimbria**	F(3,45)=2.957, p<0.042				p<0.067		p<0.079
	**Hippocampus**	F(3,45)=2.263, p<0.094						
	**Internal capsule**	F(3,45)=0.516 p<0.673						
	**Optic tract**	F(3,45)=1.115, p<0.353						
	**Thalamus**	$\chi^2(3)$=6.739, p<0.081						
**GLUT-1 Intensity**	**Anterior commissure**	$\chi^2(3)$=7.416, p<0.060						
	**Basal ganglia**	F(3,45)=5.195, p<0.004		p<0.064				p<0.003
	**Corpus callosum**	F(3,45)=1.954, p<0.134						
	**Cortex**	F(3,45)=4.084, p<0.012	p<0.006	p<0.066				
	**External capsule**	F(3,45)=1.413, p<0.251						
	**Fimbria**	F(3,45)=0.844, p<0.477						
	**Hippocampus**	F(3,45)=3.945, p<0.014	p<0.007					
	**Internal capsule**	F(3,45)=3.257, p<0.030						p<0.068
	**Optic tract**	F(3,45)=1.401, p<0.255						
	**Thalamus**	F(3,45)=1.534, p<0.219						

Supplementary Table S15. Summary statistics of Diffusion tensor imaging analysis. Abbreviations: high-fat diet (HFD), branched-chain amino acids (BCAA), fractional anisotropy (FA), mean diffusivity (MD).

Parameter		Effects	Baseline → 3.5 months		3.5 months → 6 months	Baseline → 6 months	
			p-value	relative change (%)	p-value	p-value	relative change (%)
FA	Grey Matter	Time effect all groups	↑F(1,32)=6.592, p<0.015	102.1		↑F(1,32)=12.849 p<0.001	104.3
	White Matter	Time effect all groups	↑F(1,32)=29.660, p<0.001	104.1		↑F(1,32)=18.617 p<0.001	103.1
	Auditory Cortex	Time effect all groups					
	Basal Ganglia	Time effect all groups	↑F(1,32)=5.315 p<0.028	102.8		↑F(1,32)=12.853, p<0.001	104.8
	Corpus Callosum	Time effect all groups	↑F(1,32)=38.500, p<0.001	105.3	↑F(1,32)=3.472, p<0.072	↑F(1,32)=17.916, p<0.001	103.9
		HFD+exercise > HFD				p<0.043	
	Fornix	Time effect all groups	↑F(1,32)=31.807 p<0.001	104.7		↑F(1,32)=16.085, p<0.001	103.6
		HFD+exercise > HFD				p<0.018	
	Hippocampus	Time effect all groups					
	Motor Cortex	Time effect all groups					
	Optic Tract	Time effect all groups	↑F(1,32)=30.483, p<0.001	105.5		↑F(1,32)=48.166, p<0.001	106.7
	Somatosensory Cortex	Time effect all groups					
MD	Grey Matter	Time effect all groups					
	White Matter	Time effect all groups	↓F(1,32)=3.950, p<0.055	99.2	↑F(1,32)=3.712, p<0.063		
	Auditory Cortex	Time effect all groups				↓F(1,32)=3.828, p<0.059	98.1
	Basal Ganglia	Time effect all groups	↓F(1,32)=9.922, p<0.004	98.6		↓F(1,32)=6.572, p<0.015	98.7
	Corpus callosum	Time effect all groups					
		Time effect HFD group			↓F(1,11)=7.644, p<0.018		
	Fornix	Time effect all groups					
		Time effect HFD group			↓F(1,11)=9.389, p<0.011		
		HFD+exercise < HFD	p<0.031				
		HFD+BCAA+exercise < HFD	p<0.028				
	Hippocampus	Time effect all groups					
	Motor Cortex	Time effect all groups					
	Optic Tract	Time effect all groups	↓F(1,32)=7.856, p<0.009	96.4		↓F(1,32)=12.736, p<0.001	96.1
	Somatosensory Cortex	Time effect all groups	↓F(1,32)=4.101, p<0.051	97.8		↓F(1,32)=5.286, p<0.028	97.8

Supplementary Table S16. Summary statistics of Polarized light imaging analysis. Abbreviations: high-fat diet (HFD), branched-chain amino acids (BCAA).

Parameters		Overall	HFD > Baseline (chow)	HFD+exercise > Baseline (chow)	HFD+BCAA+exercise < Baseline (chow)	HFD+exercise > HFD	HFD+BCAA+exercise > HFD	HFD+BCAA+exercise > HFD+exercise
**Dispersion**	**Anterior commissure**	χ2(3)=3.016, p<0.389						
	**Basal ganglia**	F(3,48)=1.183, p<0.326						
	**Corpus callosum**	χ2(3)=1.352, p<0.717						
	**Cortex**	F(3,48)= 3.133, p<0.034						
	**Cerebral peduncle**	χ2(3)=0.848, p<0.838						
	**External capsule**	F(3,48)=3.125, p<0.034						
	**Fimbria**	χ2(3)=3.591, p<0.309						
	**Fornix**	χ2(3)=1.503, p<0.682						
	**Hippocampus**	F(3,48)=2.914, p<0.044			p<0.027			
	**Internal capsule**	χ2(3)=2.717, p<0.437						
	**Motor cortex**	F(3,48)=1.594, p<0.203						
	**Optic tract**	χ2(3)=1.335, p<0.721						
	**Somatosensory cortex**	F(3,48)=0.805, p<0.497						
**Retardance**	**Anterior commissure**	F(3,43)=3.172, p<0.034		p<0.027				
	**Basal ganglia**	F(3,43)=0.570, p<0.148						
	**Corpus callosum**	χ2(3)=10.654, p<0.014	p<0.017					
	**Cortex**	χ2(3)=2.673, p<0.445						
	**Cerebral peduncle**	χ2(3)=4.068, p<0.254						
	**External capsule**	F(3,43)=1.163, p<0.335						
	**Fimbria**	χ2(3)=14.355, p<0.002	p<0.017	p<0.023				
	**Fornix**	F(3,43)=2.536, p<0.069						
	**Hippocampus**	χ2(3)=7.455, p<0.059						
	**Internal capsule**	F(3,43)=0.951, p<0.424						
	**Motor cortex**	χ2(3)=5.612, p<0.132						
	**Optic tract**	χ2(3)=10.888, p<0.012				p<0.043	p<0.079	
	**Somatosensory cortex**	F(3,43)=1.439, p<0.245						

Supplementary Table S17. Summary statistics of hippocampal volume and cortical thickness analysis.

Parameter	Effects	Baseline → 3.5 months		3.5 months → 6 months	Baseline → 6 months	
		**p-value**	**relative change (%)**	**p-value**	**p-value**	**relative change (%)**
**Cortical thickness**	Time effect all groups	F(1,38)=15.188, p<0.001	95.9		F(1,38)=13.317, p<0.001	96.4
	Time effect HFD group			F(1,14)=5.648, p<0.032		
**Hippocampal volume**	Time effect all groups					

Supplementary Table S18. Summary statistics of immunohistochemical analysis of ionized calcium-binding adapter molecule (IBA-1). Abbreviations: high-fat diet (HFD), branched-chain amino acids (BCAA).

Parameters		Overall	HFD > Baseline (chow)	HFD+exercise > Baseline (chow)	HFD+BCAA+exercise > Baseline (chow)	HFD+exercise < HFD	HFD+BCAA+exercise > HFD	HFD+BCAA+exercise > HFD+exercise
**IBA-1+ count/area**	**Anterior commissure**	F(3,47)=4.049, p<0.012			p<0.057			p<0.072
	**Basal ganglia**	F(3,47)=11.561, p<0.001	p<0.001	p<0.001	p<0.001			p<0.068
	**Corpus callosum**	F(3,47)=4.893, p<0.005				p<0.062		p<0.001
	**Cortex**	F(3,47)=10.021, p<0.001	p<0.001		p<0.001	p<0.021		p<0.043
	**External capsule**	F(3,47)=2.391, p<0.081						
	**Fimbria**	F(3,47)=4.545, p<0.007			p<0.007			p<0.063
	**Hippocampus**	F(3,47)=9.737, p<0.001	p<0.001	p<0.019	p<0.001			p<0.052
	**Internal capsule**	F(3,47)=4.713, p<0.006			p<0.004			p<0.049
	**Thalamus**	F(3,47)=10.324, p<0.001	p<0.001	p<0.019	p<0.001			p<0.059
**relative IBA-1+ area**	**Anterior commissure**	F(3,47)=4.049, p<0.012			p<0.057			p<0.072
	**Basal ganglia**	F(3,47)=11.561, p<0.001	p<0.001	p<0.001	p<0.001			p<0.068
	**Corpus callosum**	F(3,47)=6.925, p<0.001			p<0.01	p<0.059		p<0.001
	**Cortex**	F(3,47)=9.315, p<0.001	p<0.001		p<0.001	p<0.024		p<0.032
	**External capsule**	F(3,47)=2.783, p<0.051						
	**Fimbria**	F(3,47)=5.290, p<0.003	p<0.027		p<0.003			p<0.08
	**Hippocampus**	F(3,47)=9.047, p<0.001	p<0.001	p<0.018	p<0.001			p<0.05
	**Internal capsule**	F(3,47)=5.258, p<0.003			p<0.002			p<0.045
	**Thalamus**	F(3,47)=11.655, p<0.001	p<0.001	p<0.006	p<0.001			p<0.069

References

1. Axer, M., et al., *High-resolution fiber tract reconstruction in the human brain by means of three-dimensional polarized light imaging.* Frontiers in neuroinformatics, 2011. **5**: p. 34.
2. Mollink, J., et al., *White matter changes in the perforant path area in patients with amyotrophic lateral sclerosis.* Neuropathology and Applied Neurobiology, 2019. **45**(6): p. 570-585.
3. Axer, M., et al., *A novel approach to the human connectome: ultra-high resolution mapping of fiber tracts in the brain.* Neuroimage, 2011. **54**(2): p. 1091-1101.
